# Chromatin Sensing by the Auxiliary Domains of KDM5C Regulates Its Demethylase Activity and Is Disrupted by X-linked Intellectual Disability Mutations

**DOI:** 10.1016/j.jmb.2022.167913

**Published:** 2022-12-07

**Authors:** Fatima S. Ugur, Mark J. S. Kelly, Danica Galonić Fujimori

**Affiliations:** 1 - Chemistry and Chemical Biology Graduate Program, 600 16th St., San Francisco, CA 94158, USA; 2 - Department of Pharmaceutical Chemistry, 600 16th St., San Francisco, CA 94158, USA; 3 - Department of Cellular and Molecular Pharmacology, 600 16th St., San Francisco, CA 94158, USA; 4 - Quantitative Biosciences Institute, University of California, San Francisco, 600 16th St., San Francisco, CA 94158, USA

**Keywords:** Histone demethylase, Nucleosome, NMR, Reader domain, Intrinsically disordered region

## Abstract

The H3K4me3 chromatin modification, a hallmark of promoters of actively transcribed genes, is dynamically removed by the KDM5 family of histone demethylases. The KDM5 demethylases have a number of accessory domains, two of which, ARID and PHD1, lie between the segments of the catalytic domain. KDM5C, which has a unique role in neural development, harbors a number of mutations adjacent to its accessory domains that cause X-linked intellectual disability (XLID). The roles of these accessory domains remain unknown, limiting an understanding of how XLID mutations affect KDM5C activity. Through *in vitro* binding and kinetic studies using nucleosomes, we find that while the ARID domain is required for efficient nucleosome demethylation, the PHD1 domain alone has an inhibitory role in KDM5C catalysis. In addition, the unstructured linker region between the ARID and PHD1 domains interacts with PHD1 and is necessary for nucleosome binding. Our data suggests a model in which the PHD1 domain inhibits DNA recognition by KDM5C. This inhibitory effect is relieved by the H3 tail, enabling recognition of flanking DNA on the nucleosome. Importantly, we find that XLID mutations adjacent to the ARID and PHD1 domains break this regulation by enhancing DNA binding, resulting in the loss of specificity of substrate chromatin recognition and rendering demethylase activity lower in the presence of flanking DNA. Our findings suggest a model by which specific XLID mutations could alter chromatin recognition and enable euchromatin-specific dysregulation of demethylation by KDM5C.

## Introduction

The methylation of lysine 4 on histone H3 is a chromatin modification found on euchromatin, where H3K4 trimethylation (H3K4me3) is present at gene promoter regions associated with active transcription, and where H3K4 monomethylation (H3K4me1) is found at active enhancer regions.^1^ While H3K4me1/2 is demethylated by the KDM1/LSD family, H3K4me1/2/3 is dynamically regulated by the KDM5/JARID1 subfamily of Jumonji histone demethylases.^2–7^ This demethylase family harbors unique auxiliary domains in addition to its catalytic domain comprised of the JmjN and JmjC segments that form a composite active site for demethylation.^8–9^ KDM5A (RBP2, JARID1A), KDM5B (PLU-1, JARID1B), KDM5C (SMCX, JARID1C), and KDM5D (SMCY, JARID1D) all contain an AT-rich interaction domain (ARID), C_5_HC_2_ zinc finger domain (ZnF), and 2–3 plant homeodomains (PHD1–3). Unique to the KDM5 family is the insertion of the ARID and PHD1 domains between the JmjN and JmjC segments of the catalytic domain, and ARID and PHD1 are required for demethylase activity *in vivo*.^4,10–13^ ARID domains are DNA binding domains, and ARID of KDM5A/B has been shown to bind to GC-rich DNA.^13–15^ PHD domains are H3K4 methylation reader domains with varying specificity towards unmethylated and methylated H3K4 states.^16–22^ PHD1 of KDM5A/B preferentially binds the unmethylated H3 tail, and this recognition of the demethylation product allosterically stimulates demethylase activity of KDM5A *in vitro*.^23–28^ In contrast, the ARID and PHD1 domains have not been extensively studied in KDM5C, which possesses a unique function in neural development and has nonredundant demethylase activity.^2,29^

KDM5C is ubiquitously expressed but has highest expression levels in the brain.^30–31^ This demethylase is important for neural development and dendrite morphogenesis, and KDM5C knockout mice have abnormal dendritic branching and display memory defects, impaired social behavior, and aggression.^2,29^ KDM5C fine-tunes the expression of neurodevelopmental genes, as gene expression levels only change less than 2 fold upon knockout of KDM5C in mice.^29,32^ KDM5C localizes to enhancers in addition to promoter regions and has been shown to also demethylate spurious H3K4me3 at enhancers during neuronal maturation.^29,32–34^ In line with its neurodevelopmental function, a number of missense and nonsense mutations that cause X-linked intellectual disability (XLID) are found throughout KDM5C.^31,35–39^ As KDM5C is located on the X-chromosome and the Y paralog KDM5D cannot compensate for its function, males with KDM5C XLID mutations are primarily affected with a range of mild to severe symptoms of limitations in cognition, memory, and adaptive behavior.^30–31,37–38,40^ Some functionally characterized mutations have been shown to reduce demethylase activity despite not occurring in the catalytic domains, and a select few mutations have been shown to not affect demethylase activity, disrupting nonenzymatic functions instead.^2,11,39,41–42^ The consequences of these XLID mutations on KDM5C at its target regions within chromatin and their impact on gene expression during neural development is not fully understood. Interestingly, a number of XLID mutations are present throughout and in between the accessory domains of KDM5C, suggesting potential disruption of their regulatory functions. The impact of these mutations on demethylase regulation is hindered by the limited understanding of the accessory domain roles in KDM5C.

Here, we sought to determine the functions of the ARID and PHD1 auxiliary domains in KDM5C and evaluate whether these functions might be disrupted by XLID mutations. We approached these questions by interrogating the recognition and demethylation of nucleosomes by KDM5C, as nucleosome substrates enable extended interactions by multiple domains of the demethylase. Our findings reveal that the ARID and PHD1 domains, as well as the linker between them, regulate nucleosome demethylation and chromatin recognition by KDM5C. We find that DNA recognition by ARID contributes to nucleosome demethylation but not nucleosome binding, which is instead driven by the unstructured linker between ARID and PHD1. In contrast, we find that PHD1 inhibits demethylation. Furthermore, we find that XLID mutations near these regulatory domains disrupt interdomain interactions and enhance affinity towards nucleosomes, resulting in nonproductive chromatin binding and inhibition of demethylation in the presence of flanking DNA. Our findings define functional roles of the ARID and PHD1 domains in the regulation of KDM5C and provide rationale for disruption of this regulation by mutations in X-linked intellectual disability.

## Results

### ARID & PHD1 region contributes to productive nucleosome demethylation

**Figure 1.**
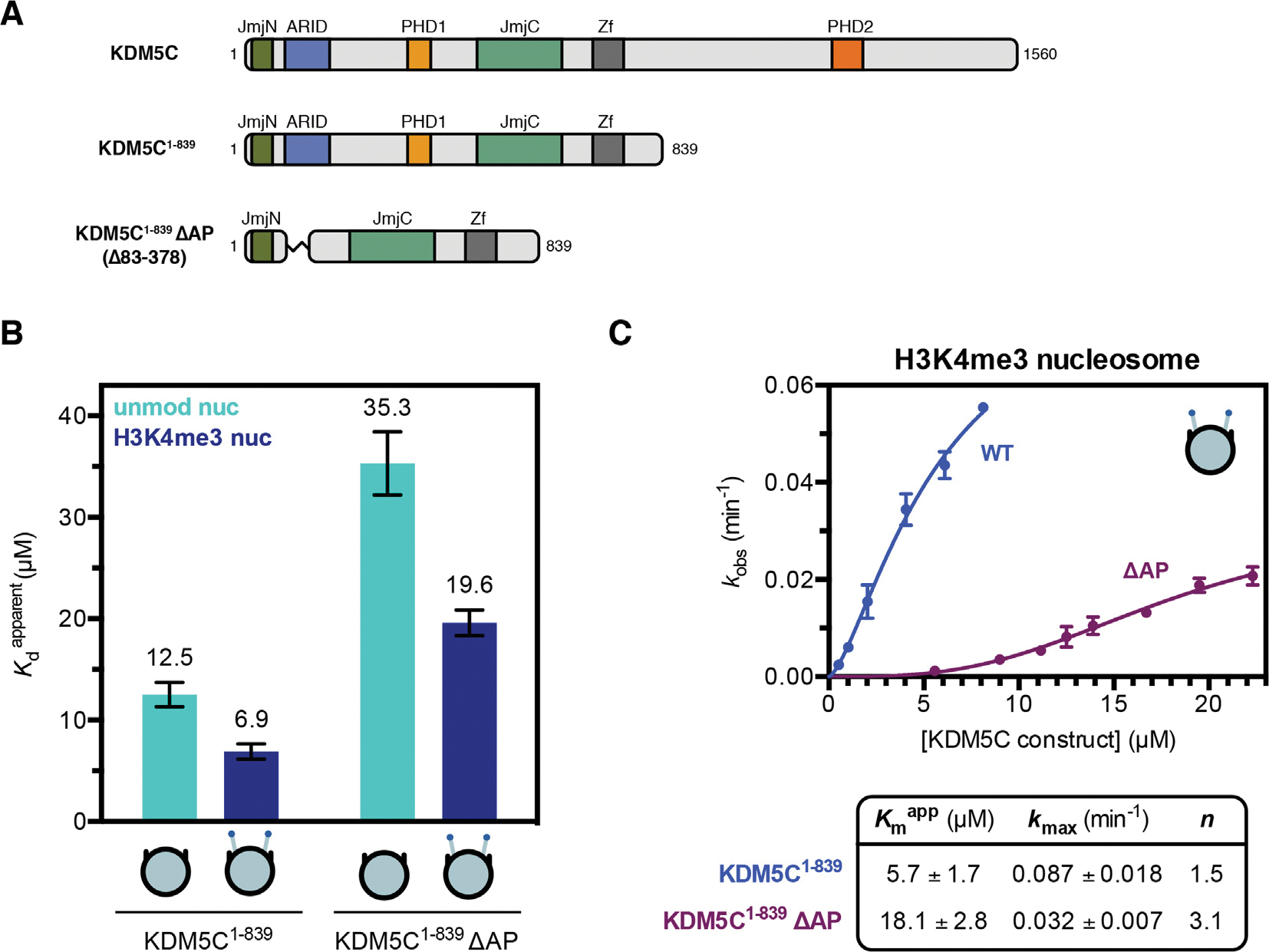
The ARID & PHD1 region of KDM5C contributes to efficient nucleosome demethylation and has a modest contribution to nucleosome binding. **(A)** Domain architecture of KDM5C and KDM5C constructs used in this study. **(B)** Unmodified and substrate nucleosome binding by KDM5C constructs with apparent dissociation constants $\left(K_{d}^{app}\right)$ measured by EMSA (binding curves in Figure S1(B)). Due to unattainable saturation of binding for the unmodified nucleosome, a lower limit for the dissociation constant is presented. **(C)** Demethylation kinetics of the H3K4me3 substrate nucleosome by KDM5C constructs under single turnover conditions (enzyme in excess of substrate). Observed rates are fit to a cooperative kinetic model, with *n* denoting the Hill coefficient. Representative kinetic traces used to determine observed demethylation rates are in Figure S1(C). All error bars represent SEM of at least three independent experiments (n ≥ 3).

Previous work has demonstrated that KDM5C is capable of demethylating H3K4me3 peptides and that the catalytic JmjN-JmjC domain and zinc finger domain are necessary for demethylase activity.^2,8,42^ To evaluate the contributions of the ARID and PHD1 domains, we sought to interrogate the recognition and demethylation of nucleosomes, given the expected interactions of these domains with DNA and histone tails, respectively. We utilized an N-terminal fragment of KDM5C containing the residues 1 to 839 necessary to monitor demethylation *in vitro* (KDM5C^1–839^), as well as an analogous construct where the ARID and PHD1 region (residues 83 to 378) is replaced by a short linker (KDM5C^1–839 ΔAP^) (Figure 1(A)).^8^ We measured binding affinities of these constructs to both unmodified and substrate H3K4me3 core nucleosomes containing 147 bp DNA by electrophoretic mobility shift assay. KDM5C binds nucleosomes with weak affinity and with approximately a two fold affinity gain towards substrate nucleosomes, with $K_{d}^{app}$ of ~7 μM for the H3K4me3 nucleosome and ~13 μM for the unmodified nucleosome (Figure 1(B)). Surprisingly, the ARID and PHD1 domains have a modest contribution to nucleosome binding, as KDM5C^1–839 ΔAP^ displays only a ~3 fold reduction in nucleosome affinity and retains the two fold preference towards the substrate nucleosome (Figure 1 (B)). The absence of a significant enhancement of nucleosome binding through ARID and PHD1 domain-mediated interactions suggests a more complex role of these domains rather than simply facilitating chromatin recruitment.

We next interrogated the demethylase activity of KDM5C towards the H3K4me3 substrate nucleosome *in vitro* by utilizing a TR-FRET based kinetic assay that detects formation of the H3K4me1/2 product nucleosome. In order to measure true catalytic rates $\left(k_{max}\right)$, demethylation was performed under single turnover conditions with enzyme in excess.^43^ We find that KDM5C^1–839^ demethylates the substrate nucleosome with an observed catalytic rate of ~0.09 min^−1^ and KDM5C^1–839 ΔAP^ with a 3-fold lower catalytic rate of 0.03 min^−1^ (Figure 1(C)), indicating that the ARID and PHD1 region contributes to productive catalysis on nucleosomes. The contribution of the ARID and PHD1 domain region towards efficient demethylation appears to be through interactions of these domains with the nucleosome, as the catalytic efficiency $\left(K_{max}/K_{mpp}^{pp}\right)$ of KDM5C^1–839 ΔAP^ relative to wild type is only 3-fold lower on the substrate H3K4me3 peptide (Figure S1(A)), as opposed to the 9-fold reduction in catalytic efficiency on the substrate nucleosome. As the ARID and PHD1 domains are poorly functionally characterized in KDM5C, we sought to next investigate the features of the nucleosome that they recognize.

### PHD1 domain inhibits KDM5C catalysis

**Figure 2.**
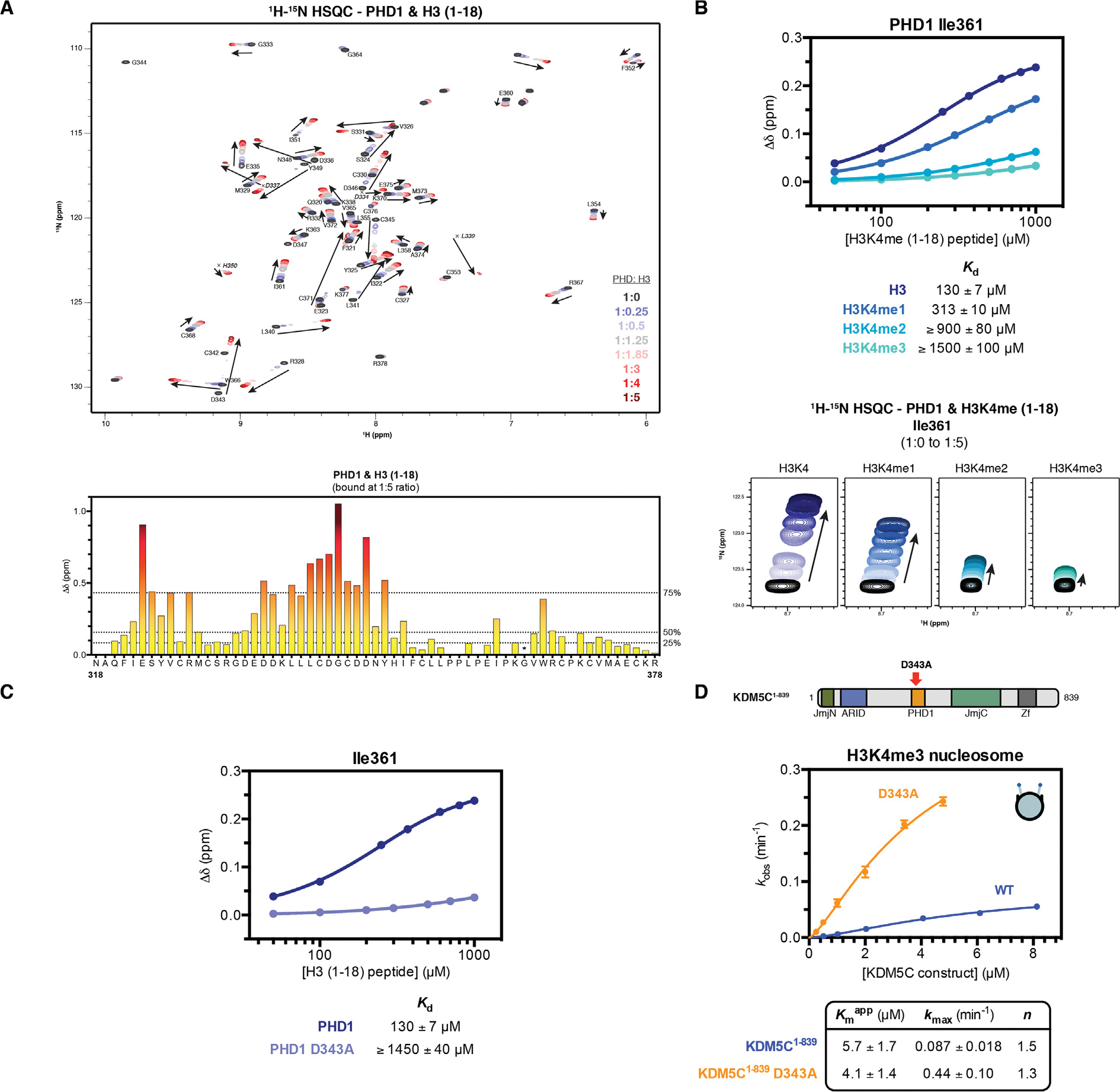
The PHD1 domain of KDM5C preferentially binds the unmodified H3 tail and has an inhibitory role towards nucleosome demethylation. **(A)** 2D ^1^H-^15^N HSQC spectra of PHD1 titrated with increasing amounts of H3 (1–18) peptide with indicated molar ratios (*top*). Backbone assignments of residues in PHD1 are labeled. Corresponding chemical shift change (Δδ) of PHD1 residues upon binding of the H3 (1–18) tail peptide at 1:5 molar ratio (PHD:peptide) (*bottom*). The chemical shift change of G364 (* denoted by asterisk) could not be determined due to broadened chemical shift when bound. Dashed lines indicate 25th, 50th, and 75th percentile rankings, and residues are colored by a gradient from unperturbed (yellow) to significantly perturbed (maroon). Perturbations colored by the gradient and mapped to homologous residues in the structure of KDM5D PHD1 are in Figure S2(C). **(B)** 2D ^1^H-^15^N HSQC of I361 in PHD1 upon titration of H3K4me0/1/2/3 (1–18) peptides (*bottom*) with dissociation constants determined from the chemical shift change (Δδ) of I361 with standard error (*top*). Due to incomplete saturation of binding, a lower limit for the dissociation constant is presented for the H3K4me2/3 peptides. Dissociation constants determined from chemical shift changes of several PHD1 residues are in Figure S2(H). **(C)** Binding of the H3 (1–18) tail peptide by PHD1 and PHD1 D343A mutant measured by NMR titration HSQC experiments. The chemical shift change (Δδ) of I361 in PHD1 was fit to obtain dissociation constants with standard error. Due to incomplete saturation of binding by the D343A mutant, a lower limit for the dissociation constant is presented. **(D)** Demethylation kinetics of the H3K4me3 substrate nucleosome by wild type and PHD1 mutant KDM5C^1–839^ under single turnover conditions. Observed rates are fit to a cooperative kinetic model, with *n* denoting the Hill coefficient. Wild type kinetic curve replotted from Figure 1(C) for comparison. Error bars represent SEM of at least three independent experiments (n ≥ 3).

The PHD1 domain of KDM5C has been previously shown to bind to H3K9me3 through peptide pull down.^2^ To interrogate the histone binding and specificity of PHD1, we purified the PHD1 domain and quantified binding to histone peptides by nuclear magnetic resonance (NMR) spectroscopy and bio-layer interferometry (BLI). We observe near identical binding between H3 and H3K9me3 tail peptides, indicating no specific binding of PHD1 towards the H3K9me3 modification (Figure S2(A)). Furthermore, we observe biphasic binding kinetics of PHD1 binding the H3 tail peptide, indicative of a two step binding mechanism (Figure S2(B)). Upon titration of the H3 tail, large chemical shift changes occur in the two-dimensional heteronuclear single quantum coherence (HSQC) NMR spectrum of a majority of assigned residues in PHD1 (Figures 2(A) and S2(C)). The observed affinity of PHD1 towards the H3 tail is surprisingly weak with a dissociation constant of 130 μM, about 100 fold weaker than the affinity of the homologous PHD1 of KDM5A towards the H3 tail (Figure 2 (B)).^25,28^ Despite this difference in affinity, PHD1 of KDM5C retains similar specificity towards the unmodified H3 tail over H3K4 methylated tail peptides as observed in the PHD1 domains of KDM5A/B (Figure 2(B)).

In order to investigate the function of PHD1 binding to the H3 tail in KDM5C catalysis, we sought to disrupt the PHD1-H3 interaction through mutagenesis. One of the largest chemical shift perturbations that occurs in PHD1 upon H3 tail binding is at the D343 residue, a residue homologous to D312 in PHD1 of KDM5A where this residue is involved in H3R2 recognition (Figure S2(D)).^44^ Similarly to PHD1 of KDM5A, we observe a dependence of histone tail binding on recognition of the H3R2 residue by PHD1 of KDM5C (Figure S2(E)). Like the mutation of D312 in KDM5A, the D343A mutation decreases the affinity of KDM5C PHD1 to the H3 tail at least 10 fold (Figure 2(C)).^25^ When introduced into the KDM5C^1–839^ enzyme, the D343A mutation does not affect the catalytic rate of H3K4me3 peptide demethylation (Figure S2(F)). Surprisingly, the D343A PHD1 mutant enzyme demethylates the H3K4me3 nucleosome more rapidly than wild type KDM5C^1–839^, with a ~5 fold increase of $k_{max}$ (Figure 2(D)). No significant change in nucleosome binding due to the D343A mutation in KDM5C^1–839^ was observed (Figure S2(G)). This data supports an inhibitory role of the PHD1 domain in nucleosome demethylation by KDM5C. This inhibitory role is in stark contrast to that observed for the PHD1 domain in KDM5A, where the PHD1 domain has a stimulatory role in catalysis.^25,28^

### ARID domain contributes to nucleosome demethylation by KDM5C

**Figure 3.**
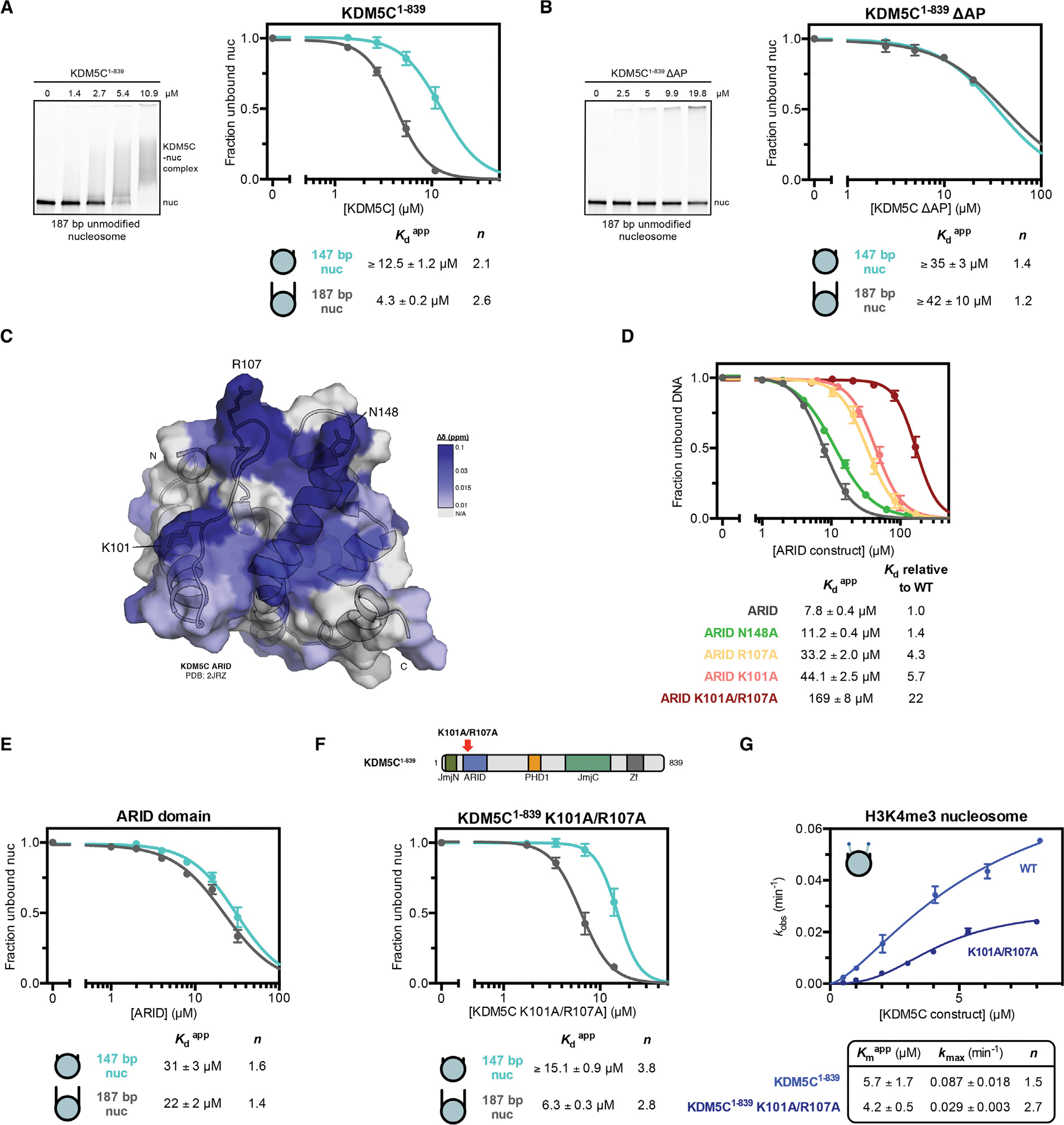
DNA recognition by the ARID domain is needed for nucleosome demethylation but not nucleosome binding by KDM5C. **(A)** Binding of KDM5C^1–839^ to unmodified nucleosomes with and without 20 bp flanking DNA. Representative gel shift of KDM5C binding to the 187 bp nucleosome (*left*). Nucleosome binding curves measured by EMSA and fit to a cooperative binding model to determine apparent dissociation constants $\left(K_{d}^{app}\right)$, with *n* denoting the Hill coefficient (*right*). Due to unattainable saturation of binding, a lower limit for the dissociation constant is presented for the unmodified core nucleosome. **(B)** Binding of KDM5C^1–839 ΔAP^ to unmodified nucleosomes with and without 20 bp flanking DNA. Representative gel shift of KDM5C ΔAP binding to the 187 bp nucleosome (*left*) and nucleosome binding curves of KDM5C^1–839 ΔAP^ (*right*). Due to unattainable saturation of binding, a lower limit for the dissociation constant is presented. **(C)** Chemical shift changes of ARID binding to 20 bp 5′ flanking DNA colored by the gradient and mapped to the KDM5C ARID structure (PDB: 2JRZ) of residues with backbone assignments in the^1^H- ^15^N HSQC spectrum. Significantly perturbed residues are labeled. **(D)** DNA (147 bp 601 core nucleosome positioning sequence) binding by ARID and ARID mutants. Binding curves were measured by EMSA and fit to a cooperative binding model to determine apparent dissociation constants $\left(K_{d}^{app}\right)$. **(E)** Nucleosome binding curves of the ARID domain binding to unmodified nucleosomes with and without 20 bp flanking DNA. **(F)** Nucleosome binding curves of ARID mutant KDM5C^1–839^ K101A/R107A binding to unmodified nucleosomes with and without 20 bp flanking DNA. **(G)** Demethylation kinetics of the H3K4me3 core substrate nucleosome by wild type and ARID mutant KDM5C^1–839^ under single turnover conditions. Observed rates are fit to a cooperative kinetic model, with *n* denoting the Hill coefficient. Wild type kinetic curve replotted from Figure 1(C) for comparison. All error bars represent SEM of at least three independent experiments (n ≥ 3).

In contrast to the inhibition of KDM5C demethylation by the PHD1 domain alone, together the ARID and PHD1 domains provide catalytic enhancement on nucleosomes (Figure 1 (C)). We hypothesize that this effect may be due to the ability of the ARID domain to interact with DNA, similarly to the previously demonstrated DNA recognition by the ARID domains of KDM5A/B.^13–15^ To test this hypothesis, we interrogated binding of KDM5C^1–839^ towards nucleosomes containing 20 bp flanking DNA on both ends (187 bp nucleosome). Strikingly, we observe a 3-fold gain in affinity towards the 187 bp nucleosome compared to the core (147 bp) nucleosome (Figure 3 (A)), demonstrating that KDM5C is capable of recognizing flanking DNA. KDM5C^1–839 ΔAP^ has similar affinity towards both the flanking DNA-containing and core nucleosome (Figure 3(B)), indicating that the ARID and PHD1 region is responsible for the recognition of flanking DNA.

To further analyze DNA recognition, we purified the KDM5C ARID domain and interrogated its ability to bind the flanking DNA present in the 187 bp nucleosome used in this study. We find that the ARID domain binds the 5′ flanking DNA fragment, with a dissociation constant of 10 μM (Figure S3(A)). Minimal binding was observed for the 3′ flanking DNA fragment (Figure S3(A)), suggesting sequence specificity in DNA binding by ARID. We utilized NMR spectroscopy to identify the residues of the ARID domain involved in DNA binding. Previously determined assignments for the ARID domain were reliably transferred to a majority of resonances observed in the ^1^H-^15^N HSQC of ARID, and modest chemical shift changes of select ARID residues were observed upon titration of the 5′ flanking DNA fragment (Figure S3(B,C)).^45^ The perturbed residues localize to a surface on the structure of KDM5C ARID (Figure 3(C)), with the most notable chemical shift changes at the K101, V105, E106, R107, and N148 residues.^45^

We interrogated the contributions of several identified residues, K101, R107, and N148, towards DNA binding through mutagenesis, where we tested binding to the 147 bp 601 core nucleosome positioning sequence (Figure 3(D)). We find the N148A mutation does not significantly affect DNA binding by ARID, while the K101A and R107A mutations reduce DNA binding by 4–6 fold (Figure 3(D)). A further 22-fold reduction in DNA binding was observed upon the K101A/R107A double mutation in ARID (Figure 3(D)), indicating that the K101 and R107 residues are significantly involved in DNA recognition. These residues parallel those identified in the ARID domains of KDM5A/B where the homologous residues, R112 of KDM5A and K119 & R125 of KDM5B, contribute to DNA binding, suggesting conservation of DNA binding residues in the KDM5 family.^13,15^

We next interrogated DNA binding by ARID in the context of the 147 bp and 187 bp nucleosomes. We find the ARID domain does not display a strong binding preference for the flanking DNA-containing nucleosome and instead binds both nucleosomes with a similar weak affinity (Figure 3(E)). The observed binding corresponds to a 3–4 fold reduction in affinity relative to 147 bp non-nucleosomal DNA (Figure 3(D,E)).

We then investigated the function of ARID in the context of the KDM5C enzyme towards nucleosome binding and demethylation by introducing the K101A/R107A double mutation into KDM5C^1–839^. We find that ARID mutant KDM5C^1–839^ retains a similar binding affinity as wild type KDM5C^1–839^ towards both the flanking DNA-containing and core nucleosome (Figure 3(F,A)). This indicates that the ARID domain does not contribute to nucleosome binding or to recognition of flanking DNA by KDM5C, in contrast to our original hypothesis. However, ARID mutant KDM5C^1–839^ has a reduced ability to demethylate the H3K4me3 nucleosome, with a 3-fold reduction in $k_{max}$ relative to wild type KDM5C^1–839^ (Figure 3 (G)). Reduced catalysis by the ARID mutant enzyme is only observed on the nucleosome, as the K101A/R107A double mutation does not reduce the catalytic rate of H3K4me3 peptide demethylation (Figure S3(D)). The similarity of catalytic rates of nucleosome demethylation between ARID mutant KDM5C^1–839^ and KDM5C^1–839^ ΔAP (0.029 min^−1^ and 0.032 min^−1^, respectively) implicates the ARID-DNA interaction as the significant contributor in the ARID and PHD1 region towards catalysis rather than nucleosome recognition (Figure 3(G), Figure 1(C)).

### PHD1 regulates the ability of KDM5C to recognize flanking DNA on the nucleosome

**Figure 4.**
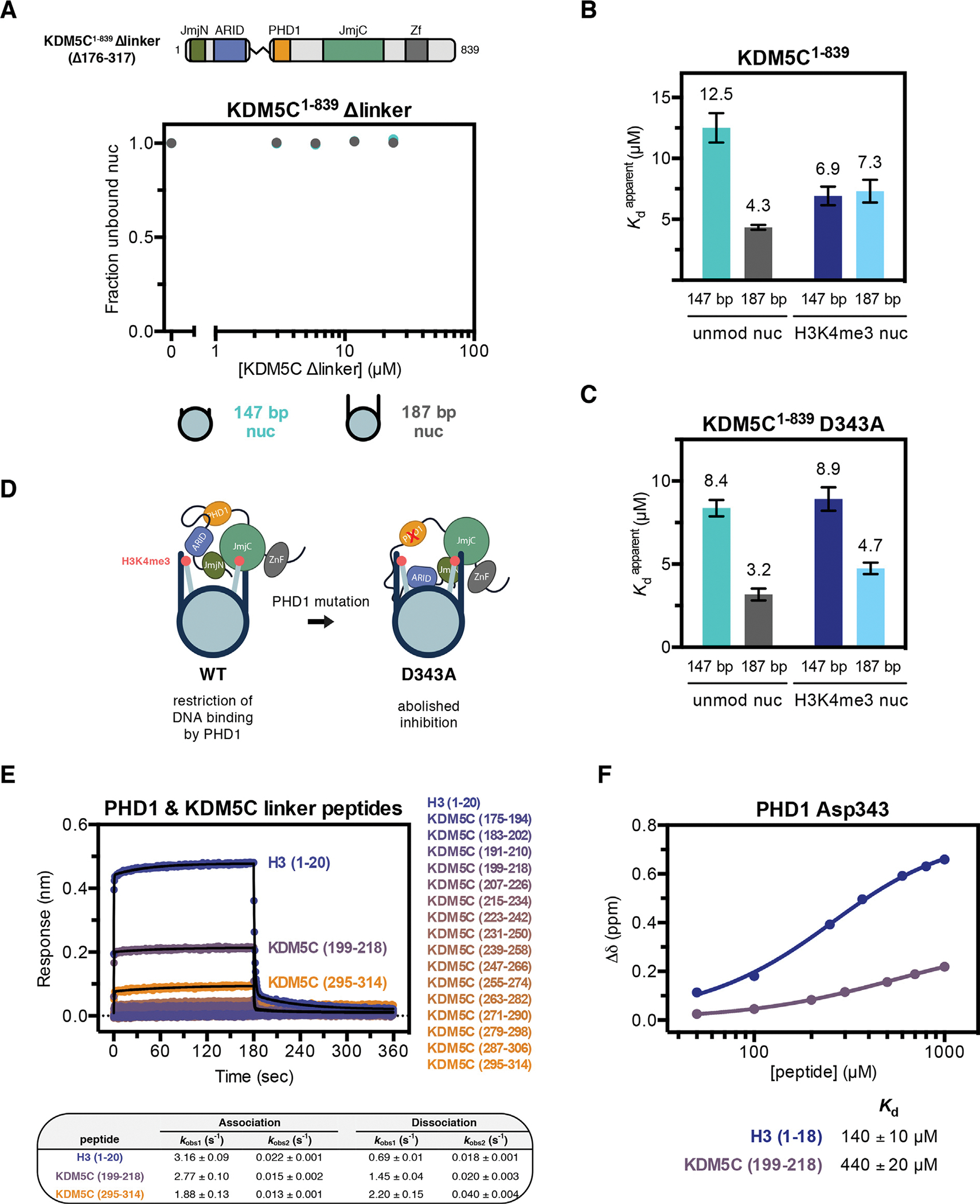
KDM5C recognizes flanking DNA in the absence of H3K4me3 due to regulation by PHD1. **(A)** Binding of KDM5C^1–839 Δlinker^ to unmodified nucleosomes with and without 20 bp flanking DNA. Nucleosome binding curves were measured by EMSA. **(B)** Nucleosome binding by KDM5C^1–839^ with apparent dissociation constants $\left(K_{d}^{app}\right)$ measured by EMSA and fit to a cooperative binding model (substrate nucleosome binding curves in Figure S4(H)). Select dissociation constants replotted from Figures 1(B) and 3(A) for comparison. Due to unattainable saturation of binding, a lower limit for the dissociation constant is presented for the unmodified core nucleosome. **(C)** Nucleosome binding by PHD1 mutant KDM5C^1–839^ D343A with apparent dissociation constants $\left(K_{d}^{app}\right)$ measured by EMSA (binding curves in Figure S4(I)). **(D)** Model for KDM5C inhibition, where PHD1 prevents flanking DNA recognition in the presence of H3K4me3, and its relief by the PHD1 mutation that disrupts the inhibition. **(E)** Binding kinetic trace of immobilized Avitag-PHD1 binding to H3 (1–20) tail peptide and KDM5C ARID-PHD1 linker fragment 20-mer peptides measured by bio-layer interferometry. Observed rates $\left(k_{\text{obs}\;}\right)$ of association and dissociation by peptides with detectable binding were obtained from fitting kinetic traces to a two phase exponential function. KDM5C linker fragment peptides have acetylated N-termini and amidated C-termini. Identified PHD1-binding KDM5C peptide sequences are KDM5C (199–218): QSVQPSKFNSYGRRAKRLQP and KDM5C (295–314): KEELSHSPEPCTKMTMRLRR. **(F)** Binding of the H3 (1–18) tail peptide and KDM5C (199–218) peptide by PHD1 measured by NMR titration HSQC experiments. The chemical shift change (Δδ) of D343 in PHD1 was fit to obtain dissociation constants with standard error. All error bars represent SEM of at least three independent experiments (n ≥ 3).

Unlike wild type (Figure 3(A)) and ARID mutant KDM5C (Figure 3(F)), KDM5C^1–839 ΔAP^ has reduced nucleosome binding and a loss in the ability to discriminate between the 147 bp and 187 bp nucleosome (Figure 3(B)). To better understand elements of KDM5C that contribute to its ability to bind DNA in the context of the nucleosome, we focused on the linker region between ARID and PHD1. The ARID-PHD1 linker region of KDM5C is the longest among KDM5 family members and contains many basic residues (Figure S4(A)). This linker region also has low conservation in the KDM5 family and is predicted to be disordered in KDM5C (Figure S4 (A,B)) [74]. We generated a construct where the linker region (residues 176 to 317) is replaced by a short (GGS)_5_ linker sequence (KDM5C^1–839 Δlinker^) (Figure 4(A)). KDM5C^1–839 Δlinker^ possesses similar catalytic efficiencies as wild type KDM5C^1–839^ on both the H3K4me3 nucleosome and H3K4me3 peptide substrate (Figure S4(C,D)), indicating that the enzyme without the ARID-PHD1 linker is functionally active. We then assessed binding of KDM5C^1–839 Δlinker^ to the 147 bp and 187 bp nucleosome and surprisingly did not detect any nucleosome binding (Figure 4(A)), suggesting that the linker region affects nucleosome and flanking DNA recognition by KDM5C.

We next interrogated recognition of flanking DNA on the nucleosome in the presence of the H3K4me3 substrate, as recognition of both could facilitate recruitment of KDM5C to its target sites in euchromatin.^29^ Intriguingly, KDM5C^1–839^ has similar binding affinity for both the core and flanking DNA-containing H3K4me3 nucleosome, with $K_{d}^{app}$ of ~7 μM, indicating no engagement of flanking DNA in the presence of the H3K4me3 substrate (Figure 4 (B)). This is in contrast to unmodified nucleosome binding, where KDM5C has a clear preference for nucleosomes with flanking DNA (Figure 4(B)).

Since KDM5C recognizes flanking DNA only in the context of the unmodified nucleosome, we considered the possibility that the ability to engage flanking DNA is coupled to binding of the H3 tail product to the PHD1 domain. To test this model, we interrogated the effect of the PHD1 D343A mutation, which abrogates H3 binding, on the recognition of flanking DNA by KDM5C. We find that PHD1 mutant KDM5C^1–839^ D343A still retains the 3-fold affinity gain towards the unmodified 187 bp nucleosome $\left(K_{d}^{\text{app}\;}=3.2\mu M\right)$ compared to the unmodified core nucleosome $\left(K_{d}^{\text{app}\;}=8.4\mu M\right)$ (Figure 4(C)). In addition, PHD1 mutant KDM5C displays a 2 fold affinity gain towards the 187 bp H3K4me3 nucleosome $\left(K_{d}^{app}=4.7\mu M\right)$, relative to the H3K4me3 core nucleosome $\left(K_{d}^{app}=8.9\mu M\right)$ (Figure 4(C)). Although modest, this improved binding demonstrates that, unlike wild type KDM5C, PHD1 mutant KDM5C can recognize flanking DNA in the presence of the H3K4me3 substrate. This observation lead us to hypothesize that, beyond disruption of H3 tail binding, the D343A mutation may also disrupt intramolecular interactions within the demethylase which restrict the ability of ARID and the ARID-PHD1 linker to interact with DNA (Figure 4(D)). This PHD1-imposed inhibition model is consistent with the strong catalytic enhancement observed with the PHD1 mutant demethylase under single turnover conditions (Figure 2(D)).

To further test this model, we examined whether PHD1 is capable of engaging in intramolecular interactions within KDM5C, which could impede its ability to interact with the H3 tail. An intramolecular interaction would necessitate that PHD1 is able to interact with ligands that do not have a free N-terminus, in contrast to typical PHD-H3 interactions.^22^ We first tested whether a free N-terminus is required for H3 tail recognition by PHD1 and find that N-terminal acetylation of the H3 tail peptide slightly reduces but does not abrogate binding by PHD1 (Figure S4(E)), indicating permissibility for recognition of an internal protein sequence. No interaction was detected between PHD1 and the ARID domain (Figure S4(F)). Using tiled peptides, we then tested binding to peptide fragments of the ARID-PHD1 linker region, each consisting of 20 amino acids with an acetylated N-terminus and amidated C-terminus. PHD1 exhibits most notable binding to the fragment of the ARID-PHD1 linker spanning residues 199–218, which contains a polybasic segment reminiscent of H3 (Figure 4 (E)). Using NMR, titration of PHD1 with the KDM5C (199–218) peptide engages a subset of residues that participate in H3 tail binding, including D343 (Figures S4(G) and 2(A)). The affinity of PHD1 towards KDM5C (199–218) is ~3-fold lower than that of the H3 tail (Figure 4(F)). These findings indicate PHD1 could interact with the ARID-PHD1 linker within KDM5C, an interaction that can be outcompeted by its H3 tail ligand.

### X-linked intellectual disability mutations alter nucleosome recognition and demethylation by KDM5C

**Figure 5.**
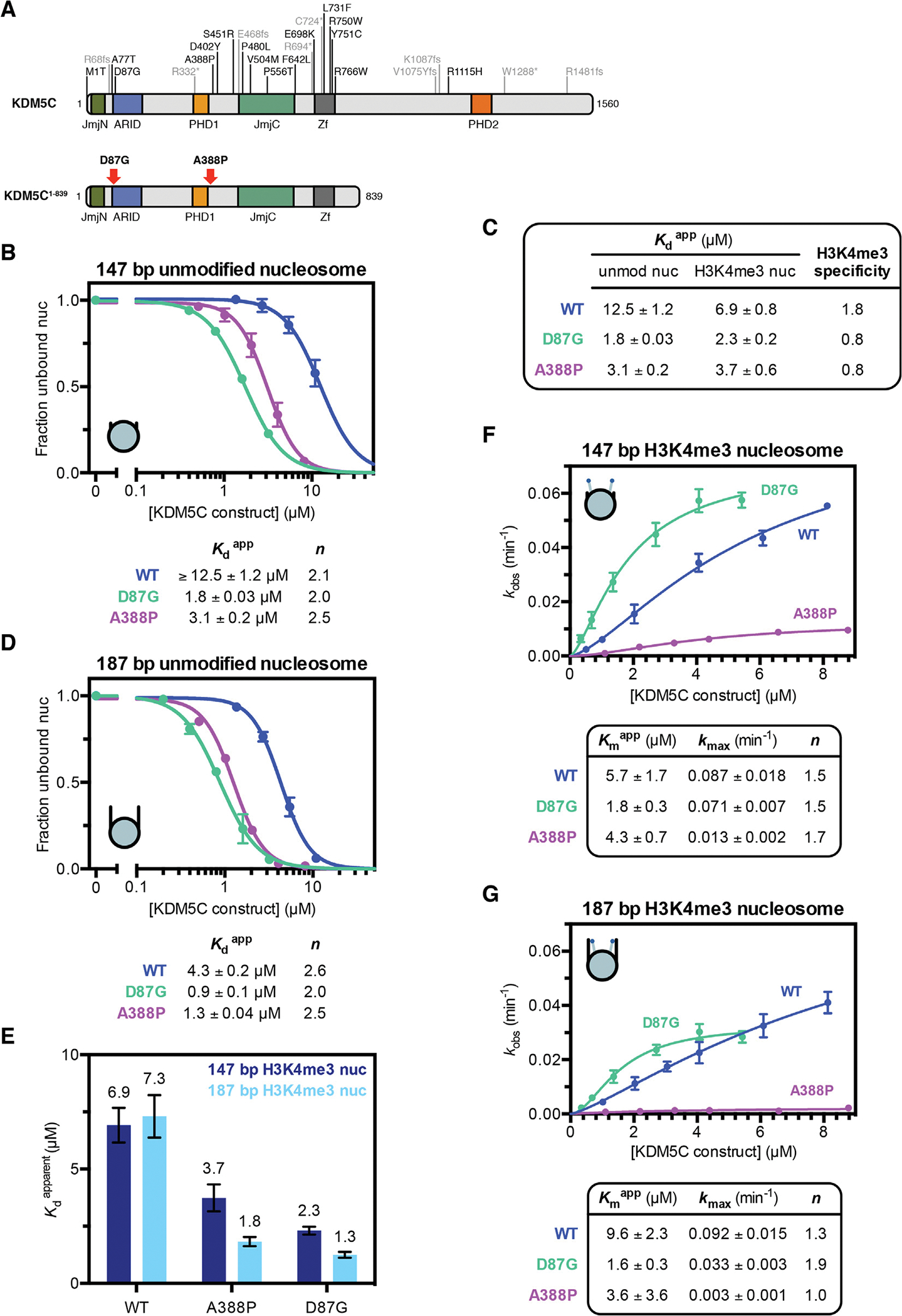
X-linked intellectual disability mutations enhance nucleosome binding by KDM5C and reduce demethylase activity in the presence of flanking DNA. **(A)** XLID mutations found in KDM5C (*top*) and the XLID mutations investigated in this study (*bottom*). **(B)** Unmodified core nucleosome binding by KDM5C^1–839^ wild type (WT), D87G, and A388P. Nucleosome binding was measured by EMSA and curves fit to a cooperative binding model to determine apparent dissociation constants $\left(K_{d}^{app}\right)$, with *n* denoting the Hill coefficient. WT binding curve replotted from Figure 3(A) for comparison. Due to unattainable saturation of binding, a lower limit for the dissociation constant is presented for WT KDM5C binding the unmodified nucleosome. **(C)** Apparent dissociation constants $\left(K_{d}^{app}\right)$ of binding by KDM5C^1–839^ WT, D87G, and A388P to unmodified and substrate core nucleosomes and resulting H3K4me3 fold binding specificity. Select dissociation constants are from Figures 1(B) and 5(B) for comparison. **(D)** Binding curves of KDM5C^1–839^ WT, D87G, and A388P binding to the unmodified 187 bp nucleosome with 20 bp flanking DNA. WT binding curve replotted from Figure 3(A) for comparison. **(E)** Binding of KDM5C^1–839^ WT, D87G, and A388P to substrate nucleosomes with and without 20 bp flanking DNA with apparent dissociation constants $\left(K_{d}^{app}\right)$ measured by EMSA (binding curves in Figure S5(C)). Select dissociation constants are replotted from Figures 4(B) and 5(C) for comparison. **(F)** Demethylation kinetics of the core substrate nucleosome by KDM5C^1–839^ WT, D87G, and A388P under single turnover conditions. Observed rates are fit to a cooperative kinetic model, with *n* denoting the Hill coefficient. Wild type kinetic curve replotted from Figure 1(C) for comparison. **(G)** Demethylation kinetics of the 187 bp substrate nucleosome by KDM5C^1–839^ WT, D87G, and A388P under single turnover conditions. All error bars represent SEM of at least three independent experiments (n ≥ 3).

Our proposed regulatory model provides a mechanistic framework for querying the effects of mutations in KDM5C that cause XLID (Figure 5 (A)). Specifically, we sought to investigate the D87G and A388P mutations found at the beginning of ARID and immediately downstream of PHD1, respectively. The D87G mutation, associated with mild intellectual disability, has been demonstrated to have no effect on global H3K4me3 levels *in vivo*.^42^ The A388P mutation, associated with moderate intellectual disability, has also been shown to have no effect on global H3K4me3 levels *in vivo* but has been reported to reduce demethylase activity *in vitro*.^2,46^ We initially interrogated nucleosome binding by KDM5C^1–839^ D87G and A388P. Strikingly, relative to wild type KDM5C^1–839^, we observe 4–7 fold enhanced binding of the XLID mutants to the unmodified core nucleosome (Figure 5(B)), suggesting that these mutations enable enhanced nucleosome engagement. The ARID and PHD1 region is required for this enhanced nucleosome binding, as there is no gain in nucleosome affinity due to the A388P mutation when the ARID and PHD1 region is removed (Figure S5(A)). Importantly, the gain in nucleosome affinity of the XLID mutants is more prominent on the unmodified core nucleosome than the substrate H3K4me3 core nucleosome, resulting in loss of binding specificity towards H3K4me3 by KDM5C due to the D87G and A388P mutations (Figure 5 (C)).

As the XLID mutations cause an overall affinity gain towards both unmodified and substrate nucleosomes, we reasoned that the recognition of the shared common epitope of DNA, rather than the H3 tail, is altered in the mutants. Indeed, relative to wild type KDM5C^1–839^, we observe a similar 3–5 fold gain in affinity by the XLID mutants towards the 187 bp unmodified nucleosome with flanking DNA, with both D87G and A388P mutants converging to a high nucleosome affinity of $K_{d}^{app}$ ~1 μM (Figure 5(D)). As flanking DNA recognition by KDM5C appears to be regulated by PHD1 (Figure 4(C)), we further interrogated recognition of the 187 bp substrate nucleosome by the D87G and A388P mutants. Both KDM5C^1–839^ D87G and A388P are capable of recognizing flanking DNA in the presence of H3K4me3, with a ~2 fold gain in affinity towards the 187 bp H3K4me3 nucleosome over the H3K4me3 core nucleosome (Figure 5(E)). These findings suggest that, similarly to the D343A PHD1 mutation (Figure 4(C)), the XLID mutations may disrupt the PHD1-mediated inhibition of DNA binding. Our findings are consistent with the model that these XLID mutations are altering the ARID and PHD1 region to relieve the inhibition of DNA binding, enabling unregulated binding to the nucleosome.

We next measured the demethylase activity of KDM5C^1–839^ D87G and A388P towards the H3K4me3 core nucleosome substrate. Despite these XLID mutants sharing similar enhanced nucleosome binding, their effects on nucleosome demethylation differ. The A388P mutation impairs KDM5C catalysis $\left(k_{max}\right)$ by ~7 fold, while the D87G mutation increases catalytic efficiency $\left(k_{max}/K_{m}^{app}\right)$ ~3 fold through an enhanced $K_{m}^{app}$, indicating both nonproductive and productive KDM5C states caused by these mutations (Figure 5(F)). The reduced demethylase activity caused by the A388P mutation is consistent with previous findings of reduced *in vitro* demethylation, with the 7-fold reduction we observe on nucleosomes exceeding the previously reported 2-fold reduction on substrate peptide.^2^ The reduced demethylase activity due to the A388P mutation might be caused by impairment of the composite catalytic domain, as we observe reduced demethylase activity in A388P mutant KDM5C^1–839^ ΔAP (Figure S5(B)). In contrast, the D87G mutation does not appear to affect the catalytic domain, and instead the improved catalytic efficiency reflects the enhancement in nucleosome binding.

Unlike wild type KDM5C, these XLID mutants recognize flanking DNA in the presence of H3K4me3, prompting us to measure demethylase activity on the 187 bp H3K4me3 nucleosome. Interestingly, while catalysis by the wild type enzyme is only slightly reduced, we find that addition of flanking DNA to the substrate nucleosome results in strong inhibition of catalysis by KDM5C^1–839^ A388P, with a 5-fold reduction in $k_{max}$ relative to the core substrate nucleosome (Figure 5(G)). Addition of flanking DNA also reduces catalysis by KDM5C^1–839^ D87G, although to a lesser degree of ~2 fold (Figure 5(G)). Despite lower maximal catalysis $\left(k_{max}\right)$ of the D87G mutant relative to wild type KDM5C^1–839^ in the presence of flanking DNA, the D87G mutant is still ~2 fold more efficient $\left(k_{max}/K_{m}^{app}\right)$ due to its enhanced nucleosome binding. Regardless, enhanced DNA recognition caused by the XLID mutations results in a reduction in the catalytic rate of H3K4me3 demethylation of nucleosomes with flanking DNA compared to core nucleosomes.

## Discussion

Different reader and regulatory domains within chromatin binding proteins and modifying enzymes influence their activity and substrate specificity by recognizing distinct chromatin states through distinguishing features on the nucleosome and surrounding DNA. Emerging structural studies of chromatin modifying enzymes in complex with nucleosomes have highlighted these multivalent interactions, with increasing observations of interactions with DNA contributing to nucleosome engagement by histone modifying enzymes.^47–59^ Despite the unique insertion of the ARID and PHD1 reader domains in the composite catalytic domain, the function of accessory domains within the KDM5 demethylase family has not been explored on nucleosomes. Here, we describe a hierarchy of regulation by these domains by investigating nucleosome recognition and demethylation in KDM5C, a unique member of the KDM5 family involved in regulation of neuronal gene transcription. We find that there are opposing roles of the ARID and PHD1 domains, with DNA recognition by ARID providing a beneficial interaction for nucleosome demethylation and regulation by PHD1 inhibiting nucleosome recognition and demethylation. We further demonstrate that DNA recognition is regulated by the PHD1 domain through its interaction with the ARID-PHD1 linker, allowing for sensing of the H3K4me3 substrate. These regulatory interactions are disrupted by the D87G and A388P XLID mutations adjacent to the ARID and PHD1 domains, resulting in enhanced DNA binding and loss of H3K4me3 specificity. As enhanced flanking DNA recognition by XLID mutants is detrimental to demethylase activity, our findings suggest dysregulation of KDM5C demethylation at euchromatic loci, where this enzyme predominantly functions.^29,32^

**Figure 6.**
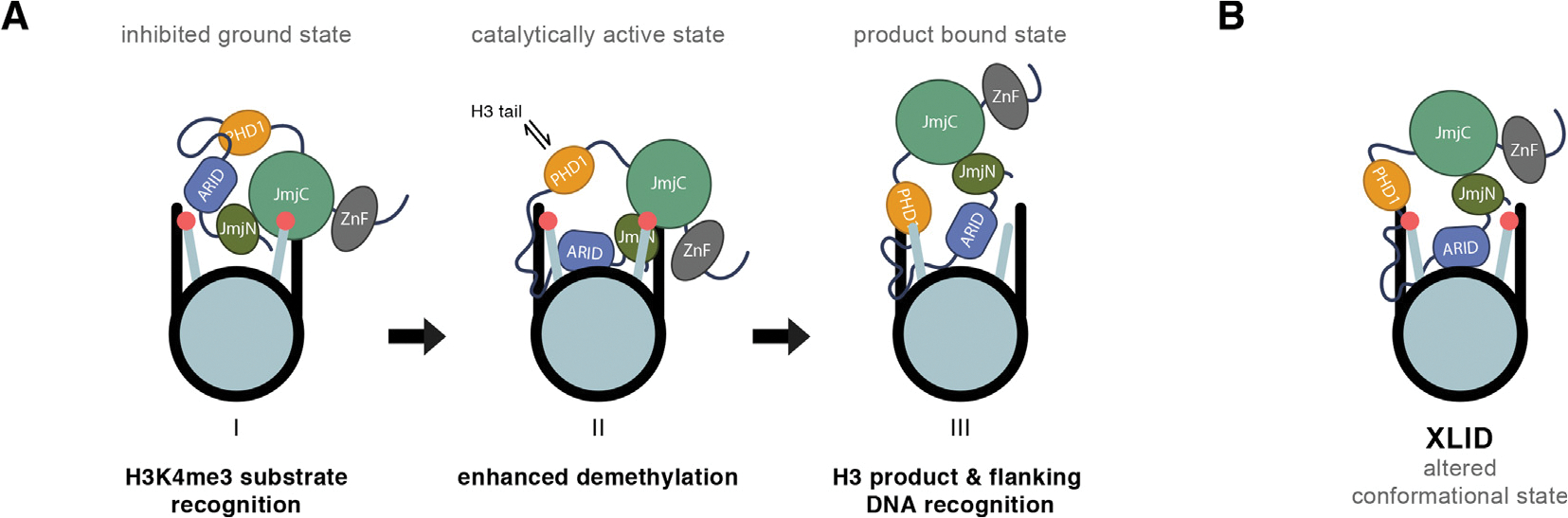
Model of KDM5C regulation by the ARID-linker-PHD1 region and KDM5C dysregulation by XLID mutations. **(A)** KDM5C recognizes H3K4me3 and binds to substrate nucleosomes through the catalytic domain (*pre-catalytic and inhibited ground state I*). DNA binding in the presence of H3K4me3 is attenuated due to an inhibitory role of PHD1 on DNA recognition. During demethylation, ARID makes transient interactions with nucleosomal DNA to orient the catalytic domain towards the H3K4me3 tail for efficient demethylation. H3 tail binding to PHD1 releases the PHD1 interaction constraining the ARID-PHD1 linker and ARID domain, enabling ARID interactions with DNA to further enhance demethylation (*catalytically active state II*). After demethylation, binding of the product H3 tail to PHD1 enables flanking DNA binding by the ARID-PHD1 linker region (*post-catalytic and product bound state III*). **(B)** Proposed altered conformational state of the ARID and PHD1 region in KDM5C due to XLID mutations in this region disrupting hypothesized intramolecular interactions.

Our findings of KDM5C nucleosome recognition and demethylation can be best explained by a regulatory model where PHD1 controls DNA recognition (Figure 6(A)). In the ground state, binding of the ARID-PHD1 linker to PHD1 restricts the ability of the enzyme to interact with DNA, attenuating catalysis (state I). Release of the PHD1-imposed constraint on the ARID-PHD1 linker and ARID domain enables improved interaction with DNA, leading to faster catalysis (state II). In our experiments, the D343A PHD1 mutation was used as a mechanistic probe to release the PHD1-imposed restriction on DNA binding. In the context of chromatin, this release of inhibition could be achieved through binding of the H3 tail to PHD1, allowing for the regulation of demethylation by the surrounding chromatin environment. Formation of the demethylated H3 product, and its binding to PHD1, further reinforces an interaction of KDM5C with chromatin by enabling flanking DNA recognition, possibly through the ARID-PHD1 linker region (state III). Alternatively, the PHD1 domain could act directly on the catalytic domains to impair productive substrate nucleosome engagement.

Intriguingly, we observe cooperativity (Hill coefficients >1) in nucleosome binding and demethylation (Figures 1(C), and S1(B)). In addition, cooperativity occurs in peptide demethylation by wild type KDM5C^1–839^ but not by KDM5C^1–839 ΔAP^ under single turnover conditions (Figure S1(A)), suggesting that cooperativity might arise both from the state of KDM5C and from the nucleosome possessing two H3 tails.

Our finding of the beneficial role of the ARID domain towards KDM5C catalysis on nucleosomes can be rationalized by favorable transient interactions of the ARID domain with nucleosomal DNA to better orient the catalytic domains for demethylation and could make the substrate H3K4me3 more accessible through disrupting histone tail-DNA interactions.^60–63^ This is supported by the previous observation that the ARID domain of KDM5C is required for its demethylase activity *in vivo* but not for its chromatin association.^11^ This role of the ARID domain in productive nucleosome demethylation may be conserved within the KDM5 family, as the ARID domain has also been found to be required for *in vivo* demethylation by KDM5A/B and the Drosophila KDM5 homolog Lid.^4,10,12–13^ The ARID domain may be required for nucleosome demethylation in order to displace the H3K4me3 tail from interacting with DNA, making it accessible for engagement by the catalytic domain. This histone tail displacement function has been proposed for DNA binding reader domain modules and for the LSD1/CoREST complex, where the SANT2 domain interacts with nucleosomal DNA to displace the H3 tail for engagement by the LSD1 active site.^55,63–65^

In contrast to the beneficial role of the ARID domain, we observe an unexpected inhibitory role of PHD1 towards KDM5C demethylation on nucleosomes. This finding suggests differential regulation by PHD1 in the KDM5 family, as PHD1 binding has a stimulatory role towards *in vitro* demethylation in KDM5A/B, and PHD1 has been previously shown to be required for demethylase activity *in vivo* for KDM5B and Lid.^4,10,25–26,28^ Our data suggests this inhibitory role is mediated by the ability of PHD1 to inhibit KDM5C’s engagement of DNA on the nucleosome (Figure 6(A)). With weak affinity and indifference for a free N-terminus (Figure S4(E)), ligand recognition by PHD1 in KDM5C is strikingly different from that observed for the PHD1 domains in KDM5A and KDM5B. While further work is needed to identify how PHD1 restricts DNA binding, our findings indicate that this could be achieved through an interaction between PHD1 and the unstructured ARID-PHD1 linker region, possibly mediated by the basic residues within the linker. This unique ARID-PHD1 linker (Figure S4 (A)) may contribute to distinct regulation by PHD1 in KDM5C. Although we are unable to directly test the effect of H3 tail binding to PHD1 on DNA recognition due to the low affinity regime, we hypothesize that the resulting binding could release inhibition, allowing for the regulation of KDM5C activity by different chromatin environments. As a consequence, H3 tail binding by PHD1 might stimulate demethylation, as observed upon PHD1 binding in KDM5A/B, through a mechanistically distinct relief of negative regulation in KDM5C (Figure 6(A)).

Unlike the ARID domain, whose DNA recognition is needed for nucleosome demethylation but not nucleosome binding, the ARID-PHD1 linker region contributes towards nucleosome binding but does not appear to contribute to demethylation by KDM5C. Surprisingly, we observe diminished nucleosome binding upon deletion of the ARIDPHD1 linker as opposed to a ~3-fold decrease in nucleosome binding upon deletion of the entire ARID and PHD1 region. While the molecular basis for these effects requires further studies, this observed discrepancy could result from the ARID and PHD1 domains affecting nucleosome binding by the catalytic and zinc finger domains of KDM5C. Our findings add to the reports of intrinsically disordered regions as functional elements within chromatin binding proteins.^66–69^

Unexpectedly, KDM5C recognizes flanking DNA around the nucleosome in the presence of the unmodified H3 tail but not in the presence of the H3K4me3 substrate. Linker DNA recognition may serve to retain KDM5C at its target promoter and enhancer sites within open chromatin after demethylation. It may also enable processive demethylation of adjacent nucleosomes in euchromatin by KDM5C. Interestingly, the recognition of linker DNA has been observed in the mechanistically unrelated H3K4me1/2 histone demethylase LSD1/KDM1A, where demethylase activity is in contrast stimulated by linker DNA.^47,70^ The H3K36me1/2 demethylase KDM2A is also capable of recognizing linker DNA, where it is specifically recruited to unmethylated CpG islands at gene promoters through its ZF-CxxC domain.^71–72^ These findings suggest that recognition of the chromatin state with accessible linker DNA may be utilized by histone modifying enzymes that function on euchromatin. While the sequence specificity of linker DNA recognition requires further investigation, it is evident that the sensing of the H3K4me3 substrate tail by KDM5C is preferred over recognition of linker DNA, a feature accessible in open chromatin. This observed hierarchy, coupled with KDM5C’s overall weak affinity towards nucleosomes and dampened demethylase activity due to regulation by PHD1, suggests tunable demethylation by KDM5C. Thus, this multi-domain regulation might serve to establish H3K4me3 surveillance through KDM5C-catalyzed demethylation, which is well suited for the physiological role of this enzyme in fine tuning gene expression through H3K4me3 demethylation at enhancers and promoters of genes, as well as its role in genome surveillance by preventing activation of non-neuronal genes in adult neurons.^29,32^

Our findings show that the regulation of DNA recognition by KDM5C is disrupted by the D87G and A388P XLID mutations adjacent to the ARID and PHD1 domains, such that nucleosome binding is significantly enhanced, H3K4me3 specificity is lost, and demethylase activity is sensitized to inhibition by linker DNA. The location of these mutations lends support to our model, where the XLID mutations relieve the inhibition of DNA recognition, enabling enhanced nucleosome binding irrespective of the methylation status of the nucleosome, by altering the conformational state of the ARID and PHD1 region (Figure 6(B)). Beyond disruption of histone demethylase activity, our findings suggest an additional mechanism of dysregulation of KDM5C in XLID, that of enhanced nonproductive chromatin engagement and differential dysregulation of demethylation at different loci depending on the accessibility of linker DNA. Despite the reduced *in vitro* activity of KDM5C due to the A388P mutation, global H3K4me3 levels are unaffected with human KDM5C A388P *in vivo*.^2,46^ In contrast, increased global H3K4me3 levels have been observed in a Drosophila intellectual disability model with A512P mutant Lid, signifying that further work is needed to profile H3K4me3 levels at genomic target regions affected by XLID mutations in human KDM5C.^73^ Furthermore, we observe that the demethylase activity of KDM5C D87G varies relative to wild type depending on the presence of flanking DNA, which might account for the unaffected global H3K4me3 levels previously observed with this D87G mutation.^42^ Our findings suggest that the chromatin environment, in particular the presence of accessible linker DNA, could govern altered demethylation and nonproductive chromatin recognition by KDM5C in XLID. Euchromatin-specific dysregulation of KDM5C demethylation might account for the hard-to-reconcile discrepancies between reported *in vitro* demethylase activities of KDM5C XLID mutants and their effect on global H3K4me3 levels.

## Materials and Methods

### Generation of KDM5C constructs

Human KDM5C gene was obtained from Harvard PlasmID (HsCD00337804) and Q175 was inserted to obtain the canonical isoform (NP_004178.2). KDM5C residues 1 to 839 were cloned into a pET28b His-Smt3 vector to produce 6xHis-SUMO-KDM5C and was mutated by site-directed mutagenesis for point mutants. The KDM5C^1–839 ΔAP^ construct was cloned by replacing residues 83–378 with a 4xGly linker. The KDM5C^1–839 Δlinker^ construct was cloned by replacing residues 176–317 with a (GGS)_5_ linker.

### Purification of KDM5C constructs

Recombinant His-tagged SUMO-KDM5C constructs were expressed in BL21(DE3) *E. coli* in LB media containing 50 μM ZnCl_2_ and 100 μM FeCl_3_ through induction at OD_600_ ~0.6 using 100 μM IPTG followed by expression at 18 °C overnight. Collected cells were resuspended in 50 mM HEPES pH 8, 500 mM KCl, 1 mM BME, 5 mM imidazole, and 1 mM PMSF, supplemented with EDTA-free Pierce protease inhibitor tablets (Thermo Fisher Scientific) and benzonase, and lysed by microfluidizer. Lysate was clarified with ultracentrifugation and the recovered supernatant was then purified by TALON metal affinity resin (total contact time under 2 hrs) at 4 °C. The His-SUMO tag was then cleaved by SenP1 during overnight dialysis at 4 °C in 50 mM HEPES pH 7.5, 150 mM KCl, and 5 mM BME. KDM5C constructs were then purified by anion exchange (MonoQ, GE Healthcare) and subsequent size exclusion (Superdex 200, GE Healthcare) chromatography in 50 mM HEPES pH 7.5 and 150 mM KCl. Fractions were concentrated and aliquots snap frozen in liquid nitrogen for storage at −80 °C.

### Nucleosomes and DNA

Recombinant human 5′ biotinylated unmodified 147 bp mononucleosomes (16–0006), unmodified 187 bp mononucleosomes (16–2104), 5′ biotinylated H3K4me3 147 bp mononucleosomes (16–0316), and 5′ biotinylated H3K4me3 187 bp mononucleosomes (16–2316) were purchased from Epicypher, Inc., in addition to biotinylated 147 bp 601 sequence DNA (18–005). 187 bp nucleosomes contain the 20 bp sequences 5′ GGACCCTATACGCGGCCGCC and GCCGGTCGCGAACAGCGACC 3′ flanking the core 601 positioning sequence. 20 bp flanking DNA duplex fragments were synthesized by Integrated DNA Technologies, Inc. For use in binding and kinetic assays, stock nucleosomes were buffer exchanged into corresponding assay buffer using a Zeba micro spin desalting column (Thermo Scientific).

### Nucleosome and DNA binding assays

Nucleosome and DNA binding was assessed by EMSA. 100 nM nucleosomes (0.5 pmol) and various concentrations of KDM5C were incubated in binding buffer (50 mM HEPES pH 7.5, 50 mM KCl, 1 mM BME, 0.01% Tween-20, 0.01% BSA, 5% sucrose) for 1 hr on ice prior to analysis by native 7.5% PAGE. For DNA binding, 100 nM 147 bp 601 sequence DNA or 500 nM 20 bp linker DNA fragments were incubated with various concentrations of ARID. Samples were separated using pre-run gels by electrophoresis in 1xTris-Glycine buffer at 100 V for 2 hrs at 4 °C, stained using SYBR Gold for DNA visualization, and imaged using the ChemiDoc imaging system (Bio-Rad Laboratories). Bands were quantified using Bio-Rad Image Lab software to determine the fraction of unbound nucleosome to calculate apparent dissociation constants by fitting to the cooperative binding equation $Y=\left(X^{\wedge }n\right)/\left(K_{d}\wedge n+X^{\wedge }n\right)$, where X is the concentration of KDM5C, n is the Hill coefficient, and K_d_ is the concentration of KDM5C at which nucleosomes are half bound.

### Single turnover nucleosome demethylation kinetics

The demethylation of biotinylated H3K4me3 nucleosome was monitored under single turnover conditions (>10 fold excess of KDM5C over substrate) through the detection of H3K4me1/2 product nucleosome formation over time by TR-FRET of an anti-H3K4me1/2 donor with an anti-biotin acceptor reagent. Various concentrations of KDM5C were reacted with 25 nM 5′ biotinylated H3K4me3 nucleosome in 50 mM HEPES pH 7.5, 50 mM KCl, 0.01% Tween-20, 0.01% BSA, 50 μM alpha-ketoglutarate, 50 μM ammonium iron(II) sulfate, and 500 μM ascorbic acid at room temperature. 5 μL time points were taken and quenched with 1.33 mM EDTA then brought to 20 μL final volume for detection using 1 nM LANCE Ultra Europium anti-H3K4me1/2 antibody (TRF0402, PerkinElmer) and 50 nM LANCE Ultra Ulight-Streptavidin (TRF0102, PerkinElmer) in 0.5X LANCE detection buffer. Detection reagents were incubated with reaction time points for 2 hours at room temperature in 384 well white microplates (PerkinElmer OptiPlate-384) then TR-FRET emission at 665 nm and 615 nm by 320 nm excitation with 50 μs delay and 100 μs integration time was measured using a Molecular Devices SpectraMax M5e plate reader. TR-FRET was calculated as the 665/615 nm emission ratio and kinetic curves were fit to a single exponential function to determine $k_{\text{obs}\;}$ of demethylation. $k_{\text{obs}\;}$ parameters were then plotted as a function of KDM5C concentration and fit to the sigmoidal kinetic equation $Y=k_{max}{\;}^{*}X^{\wedge }n/\left(K_{\text{half}\;}{\;}^{\wedge }n+X^{\wedge }n\right)$ using GraphPad Prism to determine $k_{max}$ and $K_{m}^{App}$ parameters of demethylation.

### Purification of PHD1 for NMR

PHD1 (KDM5C residues 318–378) was cloned into a pET28b His-Smt3 vector to express recombinant 6xHis-SUMO-PHD1 in BL21(DE3) *E. coli* in metal supplemented M9 minimal medium containing ^15^NH_4_Cl (Cambridge Isotope Laboratories). ^13^C-glucose (Cambridge Isotope Laboratories) was used in medium for expression of ^15^N, ^13^C-labeled PHD1. Expression was induced at OD_600_ ~0.6 using 1 mM IPTG for expression at 18 °C overnight. Collected cells were resuspended in 50 mM HEPES pH 8, 500 mM KCl, 5 mM BME, 10 mM imidazole, and 1 mM PMSF, supplemented with benzonase, and lysed by sonication. Lysate was clarified with ultracentrifugation and the recovered supernatant was then purified by Ni-NTA affinity resin. The His-SUMO tag was then cleaved by SenP1 during overnight dialysis at 4 °C in 50 mM HEPES pH 7.5, 150 μM KCl, 50 μM ZnCl_2_ and 10 μM BME. Cleaved His-SUMO tag and SenP1 was captured by passing through Ni-NTA affinity resin and flow-through was then purified by anion exchange (MonoQ) chromatography in starting buffer of 50 mM HEPES pH 7.5, 150 nM KCl, 50 μM ZnCl_2_ and 10 mM BME. Flow-through MonoQ fractions containing PHD1 were concentrated and aliquots snap frozen in liquid nitrogen for storage at −80 °C.

### PHD1 NMR and histone peptide NMR titrations

For backbone assignment of KDM5C PHD1, 400 μM ^15^N, ^13^C-labeled PHD1 in 50 mM HEPES pH 7.5, 50 mM KCl, 5 mM BME, 50 μM ZnCl_2_, and 5% D_2_O was used to perform 3D triple-resonance CBCA(CO)NH and CBCANH experiments at 298 K using a 500 MHz Bruker spectrometer equipped with a cryoprobe. Triple-resonance experiments were also performed using 400 μM ^15^N, ^13^C-labeled PHD1 bound to 2 mM H3 (1–18) peptide (1:5 ratio) to assign broadened backbone residues in apo spectra. 3D spectra were processed using NMRPipe then analyzed and assigned using Ccp NMR Analysis. Out of 56 assignable residues, 54 in apo PHD1 and 53 residues in H3 bound PHD1 were assigned.

For 2D ^1^H-^15^N HSQC spectra of KDM5C PHD1, 200 μM ^15^N-labeled PHD1 in 50 mM HEPES pH 7.5, 50 mM KCl, 5 mM BME, 50 μM ZnCl_2_, and 5% D_2_O was used to obtain 2D spectra at 298 K using a 800 MHz Bruker spectrometer equipped with a cryoprobe. Chemical shift perturbation experiments were performed by obtaining HSQC spectra with increasing concentrations of histone tail peptides (GenScript) up to 1:5 molar ratio of PHD1:peptide. Data were processed using Bruker TopSpin and analyzed using Ccp NMR Analysis. Chemical shifts were scaled and calculated as $\Delta \delta =sqrt\left(\left((\Delta \delta H{)}^{\wedge }2+(\Delta \delta N/5{)}^{\wedge }2\right)/2\right)$. Chemical shift values were then plotted as a function of histone peptide concentration and fit to the quadratic binding equation $Y=(\left(X+P_{T}+K_{d}\right)-sqrt\;{\left.\left({\left(X+P_{T}+K_{d}\right)}^{\wedge }2-4^{*}P_{T}{\;}^{*}X\right)\right)}^{*}\left(Y_{max}-Y_{min}\right)/\left(2^{*}P_{T}\right)$, where X is the concentration of peptide and P_T_ is the concentration of PHD1, using GraphPad Prism to determine K_d_ values.

### Purification of ARID for NMR

ARID (KDM5C residues 73–188) was cloned into a pET28b His-Smt3 vector to express recombinant 6xHis-SUMO-ARID in BL21(DE3) *E. coli* in metal supplemented M9 minimal medium containing ^15^NH_4_Cl. Expression was induced at OD_600_ ~0.6 using 1 mM IPTG for expression at 18 °C overnight. Collected cells were resuspended in 50 mM HEPES pH 8, 500 mM KCl, 1 mM BME, 10 mM imidazole, and 1 mM PMSF, supplemented with EDTA-free Pierce protease inhibitor tablets and benzonase, and lysed by microfluidizer. Lysate was clarified with ultracentrifugation and the recovered supernatant was then purified by Ni-NTA affinity resin. The His-SUMO tag was then cleaved by SenP1 during overnight dialysis at 4 °C in 50 mM HEPES pH 7.5, 500 mM KCl, and 5 mM BME. Cleaved His-SUMO tag and SenP1 was captured by passing through Ni-NTA affinity resin and flow-through was then purified by size exclusion (Superdex 75, GE Healthcare) chromatography in 50 mM HEPES pH 7, 150 μM KCl, and 5 mM BME. Fractions were buffer exchanged into 50 mM HEPES pH 7, 50 mM KCl, and 5 mM BME then concentrated and aliquots snap frozen in liquid nitrogen for storage at −80°C.

### ARID and DNA NMR titration

For 2D ^1^H-^15^N HSQC spectra of KDM5C ARID, 100 μM ^15^N-labeled ARID in 50 mM HEPES pH 7, 50 mM KCl, 5 mM BME, and 5% D_2_O was used to obtain 2D spectra at 298 K using a 800 MHz Bruker spectrometer equipped with a cryoprobe. Chemical-shift perturbation experiments were performed by obtaining HSQC spectra with increasing concentrations of the 5′ linker DNA 20 bp fragment up to 1:1 molar ratio of ARID: DNA. For the PHD1 titration experiment, 50 μM ^15^N-labeled ARID in 50 mM HEPES pH 7, 50 mM KCl, 5 mM BME, 50 μM ZnCl_2_, and 5% D_2_O was used with increasing concentrations of PHD1 up to 1:3 molar ratio of ARID:PHD1. Data were processed using Bruker TopSpin and analyzed using Ccp NMR Analysis. Chemical shifts were scaled and calculated as $\Delta \delta =sqrt\left(\left((\Delta \delta H{)}^{\wedge }2+(\Delta \delta \right.\right.\;\left.N/5{)}^{\wedge }2\right)\;/\;2)$. Previously determined assignments (BMRB: 15348) were transferred to a majority of resonances observed in the HSQC spectra of ARID.^45^

### Purification of ARID mutants

Recombinant His-tagged SUMO-ARID mutants were expressed in BL21(DE3) *E. coli* in 2xTY media through induction at OD_600_ ~0.6 using 1 mM IPTG followed by expression at 18 °C overnight. Collected cells were resuspended in 50 mM HEPES pH 8, 500 mM KCl, 1 mM BME, 10 mM imidazole, and 1 mM PMSF, supplemented with benzonase, and lysed by sonication. Lysate was clarified with centrifugation and the recovered supernatant was then purified by Ni-NTA affinity resin. The His-SUMO tag was then cleaved by SenP1 for 2 hours at 4 °C in 50 mM HEPES pH 7, 500 mM KCl, and 5 mM BME. Cleaved His-SUMO tag and SenP1 was captured by passing through Ni-NTA affinity resin. The flow-through was buffer exchanged into 50 mM HEPES pH 7, 50 mM KCl, and 5 mM BME then concentrated and aliquots snap frozen in liquid nitrogen for storage at −80 °C.

### Single turnover peptide demethylation kinetics

The demethylation of biotinylated H3K4me3 peptide was monitored under single turnover conditions (>10 fold excess of KDM5C over substrate) through the detection of H3K4me3 substrate loss over time by TR-FRET of an anti-rabbit IgG donor, recognizing an anti-H3K4me3 rabbit antibody, with an anti-biotin acceptor reagent. Various concentrations of KDM5C were reacted with 25 nM H3K4me3 (1–21)-biotin peptide (AS-64357, AnaSpec) in 50 mM HEPES pH 7.5, 50 mM KCl, 0.01% Tween-20, 0.01% BSA, 50 μM alpha-ketoglutarate, 50 μM ammonium iron(II) sulfate, and 500 μM ascorbic acid at room temperature. 2.5 μL time points were taken and quenched with 2 mM EDTA then brought to 20 μL final volume for detection using 1:500 dilution anti-H3K4me3 antibody (05–745R, EMD Millipore), 1 nM LANCE Ultra Europium anti-rabbit IgG antibody (PerkinElmer AD0082), and 50 nM LANCE Ultra Ulight-Streptavidin (PerkinElmer TRF0102) in 0.5X LANCE detection buffer. Detection reagents were added stepwise with 30 min incubation of anti-H3K4me3 antibody and Ulight-Streptavidin with reaction time points followed by 1 hr incubation with Europium anti-rabbit antibody in 384 well white microplates (PerkinElmer OptiPlate-384). TR-FRET emission at 665 nm and at 615 nm by 320 nm excitation with 50 μs delay and 100 μs integration time was measured using a Molecular Devices SpectraMax M5e plate reader. TR-FRET was calculated as the 665/615 nm emission ratio then subject to normalization to H3K4me3 substrate signal before demethylation. Kinetic curves were fit to a single exponential function, with the plateau set to nonspecific background of H3K4me2 product detection, to determine $k_{\text{obs}\;}$ of the H3K4me3 demethylation step. $k_{\text{obs}\;}$ parameters were then plotted as a function of KDM5C concentration and fit to the sigmoidal kinetic equation $Y=k_{max}{\;}^{*}X^{\wedge }n/\left(K_{\text{half}\;}\wedge n+X^{\wedge }n\right)$ using GraphPad Prism to determine $k_{max}$ and $K_{m}{\;}^{'}$ parameters of demethylation.

### Multiple turnover peptide demethylation kinetics

A fluorescence-based enzyme coupled assay was used to detect the formaldehyde product of demethylation of H3K4me3 peptide under multiple turnover conditions (excess of substrate peptide over KDM5C). Various concentrations of H3K4me3 (1–21) substrate peptide (GenScript) were added with 1 mM alpha-ketoglutarate to initiate demethylation by ~1 μM KDM5C in 50 mM HEPES pH 7.5, 50 mM KCl, 50 μM ammonium iron(II) sulfate, 2 mM ascorbic acid, 2 mM NAD+, and 0.05 U formaldehyde dehydrogenase (Sigma-Aldrich) at room temperature. Upon initiation, fluorescence (350 nm excitation, 460 nm emission) was measured in 20 sec intervals over 30 min using a Molecular Devices SpectraMax M5e plate reader. NADH standards were used to convert fluorescence to the rate of product concentration formed. Initial rates of the first 3 min of demethylation were plotted as a function of substrate concentration and fit to the tight-binding quadratic velocity equation $Y=V_{max}{\;}^{*}\left(\left(X+E_{T}+K_{m}\right)-sqrt\left({\left(X+E_{T}+K_{m}\right)}^{\wedge }2-4^{*}E_{T}{\;}^{*}X\right)\right)/\left(2^{*}E_{T}\right)$ using GraphPad Prism to determine Michaelis-Menten kinetic parameters of demethylation.

### Histone peptide binding kinetics

Bio-layer interferometry was used to measure binding kinetics of histone peptides to biotinylated Avitag-PHD1. Avitag followed by a linker was inserted into pET28b His-Smt3-PHD1^318–378^ to generate recombinant endogenously biotinylated 6xHis-SUMO-Avitag-(GS)_2_-PHD1 through coexpression with BirA in BL21(DE3) *E. coli* in 2xTY media containing 50 μM ZnCl_2_ and 50 μM biotin. Expression was induced at OD_600_ ~0.7 using 0.4 mM IPTG for expression at 18 °C overnight. Collected cells were resuspended in 50 mM HEPES pH 8, 500 mM KCl, 5 mM BME, 10 mM imidazole, 50 μM biotin, and 1 mM PMSF, supplemented with benzonase, and lysed by sonication. Lysate was clarified with ultracentrifugation and the recovered supernatant was then purified by Ni-NTA affinity resin. The His-SUMO tag was then cleaved by SenP1 during overnight dialysis at 4 °C in 50 mM HEPES pH 8, 150 mM KCl, 50 μM ZnCl_2_ and 10 mM BME. Cleaved His-SUMO tag and SenP1 was captured by passing through Ni-NTA affinity resin and flow-through was then purified by anion exchange (MonoQ) chromatography in starting buffer of 50 mM HEPES pH 8, 150 mM KCl, 50 μM ZnCl_2_ and 10 mM BME. Flow-through MonoQ fractions containing Avitag-PHD1 were analyzed by western blotting to identify biotinylated fractions, which were then concentrated and aliquots snap frozen in liquid nitrogen for storage at −80 °C. Using the Octet Red384 system (ForteBio) at 1000 rpm and 25 °C, 100 nM Avitag-PHD1 was loaded onto streptavidin biosensors (ForteBio) for 10 min in assay buffer (50 mM HEPES pH 8, 50 mM KCl, 50 μM ZnCl_2_, 5 mM BME, and 0.05% Tween-20) followed by 120 sec baseline then association and dissociation of 100 μM peptide (GenScript) in assay buffer. Data were processed by subtracting a single reference experiment of loaded Avitag-PHD1 without peptide. A two phase exponential function was used to fit the biphasic kinetic data using Origin software. For the ARID-PHD1 linker peptides tested to bind PHD1, the KDM5C (263–282) and KDM5C (271–290) peptides were found to nonspecifically associate with biosensors, and loaded biosensors were associated with these peptides prior to obtaining their binding traces.

## Supplementary Material

SI

## Abbreviations:

XLID: X-linked intellectual disability
KDM: lysine demethylase
H3: histone 3
H3K4: histone 3 lysine 4
H3K4me1: monomethylated lysine 4 of histone 3
H3K4me2: dimethylated lysine 4 of histone 3
H3K4me3: trimethylated lysine 4 of histone 3
PHD: plant homeodomain
ARID: AT-rich interaction domain
JmjN: Jumonji N domain
JmjC: Jumonji C domain
ZnF: zinc finger domain
KDM5C^1–839^ ΔAP: KDM5C protein containing residues 1 to 839 with truncation of the ARID and PHD1 region
KDM5C^1–839^ Δlinker: KDM5C protein containing residues 1 to 839 with truncation of the linker region between ARID and PHD1
HSQC: heteronuclear single quantum coherence
EMSA: electrophoretic mobility shift assay
TR-FRET: time-resolved fluorescence resonance energy transfer
nuc: nucleosome

## References

[1] Kimura H, (2013). Histone modifications for human epigenome analysis. J. Hum. Genet. 58, 439–445. 23739122 10.1038/jhg.2013.66

[2] Iwase S, Lan F, Bayliss P, de la Torre-Ubieta L, Huarte M, Qi HH, Whetstine JR, Bonni A, , (2007). The X-linked mental retardation gene SMCX/JARID1C defines a family of histone H3 lysine 4 demethylases. Cell 128, 1077–1088. 17320160 10.1016/j.cell.2007.02.017

[3] Klose RJ, Yan Q, Tothova Z, Yamane K, Erdjument-Bromage H, Tempst P, Gilliland DG, Zhang Y, , (2007). The retinoblastoma binding protein RBP2 is an H3K4 demethylase. Cell 128, 889–900. 17320163 10.1016/j.cell.2007.02.013

[4] Yamane K, Tateishi K, Klose RJ, Fang J, Fabrizio LA, Erdjument-Bromage H, Taylor-Papadimitriou J, Tempst P, , (2007). PLU-1 is an H3K4 demethylase involved in transcriptional repression and breast cancer cell proliferation. Mol. Cell. 25, 801–812. 17363312 10.1016/j.molcel.2007.03.001

[5] Lee MG, Norman J, Shilatifard A, Shiekhattar R, (2007). Physical and functional association of a trimethyl H3K4 demethylase and Ring6a/MBLR, a polycomb-like protein. Cell 128, 877–887. 17320162 10.1016/j.cell.2007.02.004

[6] Shi Y, Lan F, Matson C, Mulligan P, Whetstine JR, Cole PA, Casero RA, Shi Y, (2004). Histone demethylation mediated by the nuclear amine oxidase homolog LSD1. Cell 119, 941–953. 15620353 10.1016/j.cell.2004.12.012

[7] Christensen J, Agger K, Cloos PAC, Pasini D, Rose S, Sennels L, Rappsilber J, Hansen KH, , (2007). RBP2 belongs to a family of demethylases, specific for tri- and dimethylated lysine 4 on histone 3. Cell 128, 1063–1076. 17320161 10.1016/j.cell.2007.02.003

[8] Horton JR, Engstrom A, Zoeller EL, Liu X, Shanks JR, Zhang X, Johns MA, Vertino PM, , (2016). Characterization of a Linked Jumonji Domain of the KDM5/JARID1 Family of Histone H3 Lysine 4 Demethylases. J. Biol. Chem. 291, 2631–2646. 26645689 10.1074/jbc.M115.698449 PMC4742734

[9] Johansson C, Velupillai S, Tumber A, Szykowska A, Hookway ES, Nowak RP, Strain-Damerell C, Gileadi C, , (2016). Structural analysis of human KDM5B guides histone demethylase inhibitor development. Nature Chem. Biol. 12, 539–545. 27214403 10.1038/nchembio.2087

[10] Li L, Greer C, Eisenman RN, Secombe J, (2010). Essential functions of the histone demethylase lid. PLoS Genet. 6, e1001221. 21124823 10.1371/journal.pgen.1001221 PMC2991268

[11] Brookes E, Laurent B, Õunap K, Carroll R, Moeschler JB, Field M, Schwartz CE, Gecz J, , (2015). Mutations in the intellectual disability gene KDM5C reduce protein stability and demethylase activity. Hum. Mol. Genet. 24, 2861–2872. 25666439 10.1093/hmg/ddv046 PMC4406297

[12] Xiang Y, Zhu Z, Han G, Ye X, Xu B, Peng Z, Ma Y, Yu Y, , (2007). JARID1B is a histone H3 lysine 4 demethylase up-regulated in prostate cancer. Proc. Natl. Acad. Sci. U. S. A. 104, 19226–19231. 18048344 10.1073/pnas.0700735104 PMC2148272

[13] Tu S, Teng Y-C, Yuan C, Wu Y-T, Chan M-Y, Cheng A-N, Lin P-H, Juan L-J, , (2008). The ARID domain of the H3K4 demethylase RBP2 binds to a DNA CCGCCC motif. Nature Struct. Mol. Biol. 15, 419–421. 18270511 10.1038/nsmb.1400

[14] Scibetta AG, Santangelo S, Coleman J, Hall D, Chaplin T, Copier J, Catchpole S, Burchell J, , (2007). Functional analysis of the transcription repressor PLU-1/JARID1B. Mol. Cell. Biol. 27, 7220–7235. 17709396 10.1128/MCB.00274-07 PMC2168894

[15] Yao W, Peng Y, Lin D, (2010). The flexible loop L1 of the H3K4 demethylase JARID1B ARID domain has a crucial role in DNA-binding activity. Biochem. Biophys. Res. Commun. 396, 323–328. 20403335 10.1016/j.bbrc.2010.04.091

[16] Lan F, Collins RE, De Cegli R, Alpatov R, Horton JR, Shi X, Gozani O, Cheng X, , (2007). Recognition of unmethylated histone H3 lysine 4 links BHC80 to LSD1-mediated gene repression. Nature 448, 718–722. 17687328 10.1038/nature06034 PMC2702779

[17] Klein BJ, Wang X, Cui G, Yuan C, Botuyan MV, Lin K, Lu Y, Wang X, , (2016). PHF20 Readers Link Methylation of Histone H3K4 and p53 with H4K16 Acetylation. Cell Rep. 17, 1158–1170. 27760318 10.1016/j.celrep.2016.09.056 PMC5125728

[18] Shi X, Hong T, Walter KL, Ewalt M, Michishita E, Hung T, Carney D, Peña P, , (2006). ING2 PHD domain links histone H3 lysine 4 methylation to active gene repression. Nature 442, 96–99. 16728974 10.1038/nature04835 PMC3089773

[19] Li H, Ilin S, Wang W, Duncan EM, Wysocka J, Allis CD, Patel DJ, (2006). Molecular basis for site-specific read-out of histone H3K4me3 by the BPTF PHD finger of NURF. Nature 442, 91–95. 16728978 10.1038/nature04802 PMC4690523

[20] Peña PV, Davrazou F, Shi X, Walter KL, Verkhusha VV, Gozani O, Zhao R, Kutateladze TG, (2006). Molecular mechanism of histone H3K4me3 recognition by plant homeodomain of ING2. Nature 442, 100–103. 16728977 10.1038/nature04814 PMC3190580

[21] Wysocka J, Swigut T, Xiao H, Milne TA, Kwon SY, Landry J, Kauer M, Tackett AJ, , (2006). A PHD finger of NURF couples histone H3 lysine 4 trimethylation with chromatin remodelling. Nature 442, 86–90. 16728976 10.1038/nature04815

[22] Sanchez R, Zhou M-M, (2011). The PHD finger: a versatile epigenome reader. Trends Biochem. Sci. 36, 364–372. 21514168 10.1016/j.tibs.2011.03.005 PMC3130114

[23] Klein BJ, Piao L, Xi Y, Rincon-Arano H, Rothbart SB, Peng D, Wen H, Larson C, , (2014). The histone-H3K4-specific demethylase KDM5B binds to its substrate and product through distinct PHD fingers. Cell Rep. 6, 325–335. 24412361 10.1016/j.celrep.2013.12.021 PMC3918441

[24] Zhang Y, Yang H, Guo X, Rong N, Song Y, Xu Y, Lan W, Zhang X, , (2014). The PHD1 finger of KDM5B recognizes unmodified H3K4 during the demethylation of histone H3K4me2/3 by KDM5B, Protein. Cell 5, 837–850. 10.1007/s13238-014-0078-4 PMC4225485 24952722

[25] Torres IO, Kuchenbecker KM, Nnadi CI, Fletterick RJ, Kelly MJS, Fujimori DG, (2015). Histone demethylase KDM5A is regulated by its reader domain through a positive-feedback mechanism. Nature Commun. 6, 6204. 25686748 10.1038/ncomms7204 PMC5080983

[26] Zhao S, Chuh KN, Zhang B, Dul BE, Thompson RE, Farrelly LA, Liu X, Xu N, , (2021). Histone H3Q5 serotonylation stabilizes H3K4 methylation and potentiates its readout. Proc. Natl. Acad. Sci. U. S. A. 118 10.1073/pnas.2016742118. PMC8017887 33526675

[27] Wang GG, Song J, Wang Z, Dormann HL, Casadio F, Li H, Luo J-L, Patel DJ, , (2009). Haematopoietic malignancies caused by dysregulation of a chromatin-binding PHD finger. Nature 459, 847–851. 19430464 10.1038/nature08036 PMC2697266

[28] Longbotham JE, Chio CM, Dharmarajan V, Trnka MJ, Torres IO, Goswami D, Ruiz K, Burlingame AL, , (2019). Histone H3 binding to the PHD1 domain of histone demethylase KDM5A enables active site remodeling. Nature Commun. 10, 94. 30626866 10.1038/s41467-018-07829-z PMC6327041

[29] Iwase S, Brookes E, Agarwal S, Badeaux AI, Ito H, Vallianatos CN, Tomassy GS, Kasza T, , (2016). A Mouse Model of X-linked Intellectual Disability Associated with Impaired Removal of Histone Methylation. Cell Rep. 14, 1000–1009. 26804915 10.1016/j.celrep.2015.12.091 PMC4749408

[30] Jensen LR, Bartenschlager H, Rujirabanjerd S, Tzschach A, Nümann A, Janecke AR, Spörle R, Stricker S, , (2010). A distinctive gene expression fingerprint in mentally retarded male patients reflects disease-causing defects in the histone demethylase KDM5C. PathoGenetics 3, 2. 20181063 10.1186/1755-8417-3-2 PMC2830949

[31] Jensen LR, Amende M, Gurok U, Moser B, Gimmel V, Tzschach A, Janecke AR, Tariverdian G, , (2005). Mutations in the JARID1C gene, which is involved in transcriptional regulation and chromatin remodeling, cause X-linked mental retardation. Am. J. Hum. Genet. 76, 227–236. 15586325 10.1086/427563 PMC1196368

[32] Scandaglia M, Lopez-Atalaya JP, Medrano-Fernandez A, Lopez-Cascales MT, Del Blanco B, Lipinski M, Benito E, Olivares R, , (2017). Loss of Kdm5c Causes Spurious Transcription and Prevents the Fine-Tuning of Activity-Regulated Enhancers in Neurons. Cell Rep. 21, 47–59. 28978483 10.1016/j.celrep.2017.09.014 PMC5679733

[33] Outchkourov NS, Muiño JM, Kaufmann K, van Ijcken WFJ, Groot Koerkamp MJ, van Leenen D, de Graaf P, Holstege FCP, , (2013). Balancing of histone H3K4 methylation states by the Kdm5c/SMCX histone demethylase modulates promoter and enhancer function. Cell Rep. 3, 1071–1079. 23545502 10.1016/j.celrep.2013.02.030

[34] Shen H, Xu W, Guo R, Rong B, Gu L, Wang Z, He C, Zheng L, , (2016). Suppression of Enhancer Overactivation by a RACK7-Histone Demethylase Complex. Cell 165, 331–342. 27058665 10.1016/j.cell.2016.02.064 PMC4826479

[35] Gonçalves TF, Gonçalves, Fintelman Rodrigues N, dos Santos JM, Pimentel MMG, Santos-Rebouças CB, (2014). KDM5C mutational screening among males with intellectual disability suggestive of X-Linked inheritance and review of the literature. Eur. J. Med. Genet. 57, 138–144. 24583395 10.1016/j.ejmg.2014.02.011

[36] Tzschach A, Lenzner S, Moser B, Reinhardt R, Chelly J, Fryns J-P, Kleefstra T, Raynaud M, , (2006). Novel JARID1C/SMCX mutations in patients with X-linked mental retardation. Hum. Mutat. 27, 389. 10.1002/humu.9420 16541399

[37] Santos C, Rodriguez-Revenga L, Madrigal I, Badenas C, Pineda M, Milà M, (2006). A novel mutation in JARID1C gene associated with mental retardation. Eur. J. Hum. Genet. 14, 583–586. 16538222 10.1038/sj.ejhg.5201608

[38] Abidi FE, Holloway L, Moore CA, Weaver DD, Simensen RJ, Stevenson RE, Rogers RC, Schwartz CE, (2008). Mutations in JARID1C are associated with X-linked mental retardation, short stature and hyperreflexia. J. Med. Genet. 45, 787–793. 18697827 10.1136/jmg.2008.058990 PMC3711528

[39] Rujirabanjerd S, Nelson J, Tarpey PS, Hackett A, Edkins S, Lucy Raymond F, Schwartz CE, Turner G, , (2010). Identification and characterization of two novel JARID1C mutations: suggestion of an emerging genotype–phenotype correlation. Eur. J. Hum. Genet. 18, 330–335. 10.1038/ejhg.2009.175. 19826449 PMC2987212

[40] Xu J, Burgoyne PS, Arnold AP, (2002). Sex differences in sex chromosome gene expression in mouse brain. Hum. Mol. Genet. 11, 1409–1419. 10.1093/hmg/11.12.1409. 12023983

[41] Vallianatos CN, Farrehi C, Friez MJ, Burmeister M, Keegan CE, Iwase S, (2018). Altered Gene-Regulatory Function of KDM5C by a Novel Mutation Associated With Autism and Intellectual Disability. Front. Mol. Neurosci. 11, 104. 29670509 10.3389/fnmol.2018.00104 PMC5893713

[42] Tahiliani M, Mei P, Fang R, Leonor T, Rutenberg M, Shimizu F, Li J, Rao A, , (2007). The histone H3K4 demethylase SMCX links REST target genes to X-linked mental retardation. Nature 447, 601–605. 17468742 10.1038/nature05823

[43] Jones BN, Quang-Dang D-U, Oku Y, Gross JD, (2008). A kinetic assay to monitor RNA decapping under single- turnover conditions. Methods Enzymol. 448, 23–40. 19111169 10.1016/S0076-6879(08)02602-5

[44] Longbotham JE, Kelly MJS, Fujimori DG, (2021). Recognition of Histone H3 Methylation States by the PHD1 Domain of Histone Demethylase KDM5A. ACS Chem. Biol. 10.1021/acschembio.0c00976. PMC8380758 33621062

[45] Koehler C, Bishop S, Dowler EF, Schmieder P, Diehl A, Oschkinat H, Ball LJ, (2008). Backbone and sidechain 1H, 13C and 15N resonance assignments of the Bright/ARID domain from the human JARID1C (SMCX) protein. Biomol. NMR Assign. 2, 9–11. 19636912 10.1007/s12104-007-9071-7

[46] Poeta L, Padula A, Lioi MB, van Bokhoven H, Miano MG, (2021). Analysis of a Set of KDM5C Regulatory Genes Mutated in Neurodevelopmental Disorders Identifies Temporal Coexpression Brain Signatures. Genes 12 10.3390/genes12071088. PMC8305412 34356104

[47] Kim S-A, Zhu J, Yennawar N, Eek P, Tan S, (2020). Crystal Structure of the LSD1/CoREST Histone Demethylase Bound to Its Nucleosome Substrate. Mol. Cell. 78, 903–914.e4. 32396821 10.1016/j.molcel.2020.04.019 PMC7275924

[48] Kasinath V, Beck C, Sauer P, Poepsel S, Kosmatka J, Faini M, Toso D, Aebersold R, , (2021). JARID2 and AEBP2 regulate PRC2 in the presence of H2AK119ub1 and other histone modifications. Science 371 10.1126/science.abc3393. PMC7993630 33479123

[49] Finogenova K, Bonnet J, Poepsel S, Schäfer IB, Finkl K, Schmid K, Litz C, Strauss M, , (2020). Structural basis for PRC2 decoding of active histone methylation marks H3K36me2/3. Elife 9 10.7554/eLife.61964. PMC7725500 33211010

[50] Worden EJ, Zhang X, Wolberger C, (2020). Structural basis for COMPASS recognition of an H2B-ubiquitinated nucleosome. Elife 9 10.7554/eLife.53199. PMC7039682 31922488

[51] Li W, Tian W, Yuan G, Deng P, Sengupta D, Cheng Z, Cao Y, Ren J, , (2021). Molecular basis of nucleosomal H3K36 methylation by NSD methyltransferases. Nature 590, 498–503. 33361816 10.1038/s41586-020-03069-8 PMC7889650

[52] Bilokapic S, Suskiewicz MJ, Ahel I, Halic M, (2020). Bridging of DNA breaks activates PARP2–HPF1 to modify chromatin. Nature 585, 609–613. 10.1038/s41586-020-2725-7. 32939087 PMC7529888

[53] Bilokapic S, Halic M, (2019). Nucleosome and ubiquitin position Set2 to methylate H3K36. Nature Commun. 10, 3795. 31439846 10.1038/s41467-019-11726-4 PMC6706414

[54] Hsu PL, Shi H, Leonen C, Kang J, Chatterjee C, Zheng N, (2019). Structural Basis of H2B Ubiquitination-Dependent H3K4 Methylation by COMPASS. Mol. Cell. 76, 712–723.e4. 31733991 10.1016/j.molcel.2019.10.013 PMC6948180

[55] Marabelli C, Marrocco B, Pilotto S, Chittori S, Picaud S, Marchese S, Ciossani G, Forneris F, , (2019). A Tail-Based Mechanism Drives Nucleosome Demethylation by the LSD2/NPAC Multimeric Complex. Cell Rep. 27, 387–399.e7. 30970244 10.1016/j.celrep.2019.03.061

[56] Poepsel S, Kasinath V, Nogales E, (2018). Cryo-EM structures of PRC2 simultaneously engaged with two functionally distinct nucleosomes. Nature Struct. Mol. Biol. 25, 154–162. 29379173 10.1038/s41594-018-0023-y PMC5805599

[57] Park SH, Ayoub A, Lee Y-T, Xu J, Kim H, Zheng W, Zhang B, Sha L, , (2019). Cryo-EM structure of the human MLL1 core complex bound to the nucleosome. Nature Commun. 10, 1–13. 31804488 10.1038/s41467-019-13550-2 PMC6895043

[58] Xue H, Yao T, Cao M, Zhu G, Li Y, Yuan G, Chen Y, Lei M, , (2019). Structural basis of nucleosome recognition and modification by MLL methyltransferases. Nature 573, 445–449. 31485071 10.1038/s41586-019-1528-1

[59] Hatazawa S, Liu J, Takizawa Y, Zandian M, Negishi L, Kutateladze TG, Kurumizaka H, (2022). Structural basis for binding diversity of acetyltransferase p300 to the nucleosome, iScience. 25, 10.1016/j.isci.2022.104563104563. PMC9218434 35754730

[60] Gatchalian J, Wang X, Ikebe J, Cox KL, Tencer AH, Zhang Y, Burge NL, Di L, , (2017). Accessibility of the histone H3 tail in the nucleosome for binding of paired readers. Nature Commun. 8, 1489. 29138400 10.1038/s41467-017-01598-x PMC5686127

[61] Morrison EA, Bowerman S, Sylvers KL, Wereszczynski J, Musselman CA, (2018). The conformation of the histone H3 tail inhibits association of the BPTF PHD finger with the nucleosome. Elife 7 10.7554/eLife.31481. PMC5953545 29648537

[62] Stützer A, Liokatis S, Kiesel A, Schwarzer D, Sprangers R, Söding J, Selenko P, Fischle W, (2016). Modulations of DNA Contacts by Linker Histones and Post-translational Modifications Determine the Mobility and Modifiability of Nucleosomal H3 Tails. Mol. Cell. 61, 247–259. 26778125 10.1016/j.molcel.2015.12.015

[63] Peng Y, Li S, Onufriev A, Landsman D, Panchenko AR, (2021). Binding of regulatory proteins to nucleosomes is modulated by dynamic histone tails. Nature Commun. 12, 5280. 34489435 10.1038/s41467-021-25568-6 PMC8421395

[64] Weaver TM, Morrison EA, Musselman CA, (2018). Reading More than Histones: The Prevalence of Nucleic Acid Binding among Reader Domains. Molecules 23 10.3390/molecules23102614. PMC6222470 30322003

[65] Pilotto S, Speranzini V, Tortorici M, Durand D, Fish A, Valente S, Forneris F, Mai A, , (2015). Interplay among nucleosomal DNA, histone tails, and corepressor CoREST underlies LSD1-mediated H3 demethylation. Proc. Natl. Acad. Sci. U. S. A. 112, 2752–2757. 25730864 10.1073/pnas.1419468112 PMC4352788

[66] Musselman CA, Kutateladze TG, (2021). Characterization of functional disordered regions within chromatin-associated proteins, iScience. 24, 102070 33604523 10.1016/j.isci.2021.102070 PMC7873657

[67] Contreras-Martos S, Piai A, Kosol S, Varadi M, Bekesi A, Lebrun P, Volkov AN, Gevaert K, , (2017). Linking functions: an additional role for an intrinsically disordered linker domain in the transcriptional coactivator CBP. Sci. Rep. 7, 4676. 28680062 10.1038/s41598-017-04611-x PMC5498717

[68] Jiao L, Shubbar M, Yang X, Zhang Q, Chen S, Wu Q, Chen Z, Rizo J, , (2020). A partially disordered region connects gene repression and activation functions of EZH2. Proc. Natl. Acad. Sci. U. S. A. 117, 16992–17002. 32631994 10.1073/pnas.1914866117 PMC7382310

[69] Keenen MM, Brown D, Brennan LD, Renger R, Khoo H, Carlson CR, Huang B, Grill SW, , (2021). HP1 proteins compact DNA into mechanically and positionally stable phase separated domains. Elife 10 10.7554/eLife.64563. PMC7932698 33661100

[70] Kim S-A, Chatterjee N, Jennings MJ, Bartholomew B, Tan S, (2015). Extranucleosomal DNA enhances the activity of the LSD1/CoREST histone demethylase complex. Nucleic Acids Res. 43, 4868–4880. 25916846 10.1093/nar/gkv388 PMC4446439

[71] Zhou JC, Blackledge NP, Farcas AM, Klose RJ, (2012). Recognition of CpG island chromatin by KDM2A requires direct and specific interaction with linker DNA. Mol. Cell. Biol. 32, 479–489. 22083960 10.1128/MCB.06332-11 PMC3255781

[72] Blackledge NP, Zhou JC, Tolstorukov MY, Farcas AM, Park PJ, Klose RJ, (2010). CpG islands recruit a histone H3 lysine 36 demethylase. Mol. Cell. 38, 179–190. 20417597 10.1016/j.molcel.2010.04.009 PMC3098377

[73] Zamurrad S, Hatch HAM, Drelon C, Belalcazar HM, Secombe J, (2018). A Drosophila Model of Intellectual Disability Caused by Mutations in the Histone Demethylase KDM5. Cell Rep. 22, 2359–2369. 29490272 10.1016/j.celrep.2018.02.018 PMC5854480

[74] Erdős G, Pajkos M, Dosztányi Z, (2021). IUPred3: prediction of protein disorder enhanced with unambiguous experimental annotation and visualization of evolutionary conservation. Nucleic Acids Res. 49, W297–W303. 34048569 10.1093/nar/gkab408 PMC8262696

